# Supplementary material for: Extensive germline-somatic interplay contributes to prostate cancer progression through HNF1B co-option of TMPRSS2-ERG
Source: Nat Commun. 2022 Nov 28;13:7320. doi: 10.1038/s41467-022-34994-z (PMC9705428; doi:10.1038/s41467-022-34994-z)
Supplement: Supplementary file 1 — Supplementary Information [file 41467_2022_34994_MOESM1_ESM.pdf]

Supplementary Information

Extensive germline-somatic interplay contributes to prostate cancer progression through HNF1B co-option of TMPRSS2-ERG

Nikolaos Giannareas[1#], Qin Zhang[1#], Xiayun Yang[1#], Rong Na[2#], Yijun Tian[3], Yuehong Yang[1], Xiaohao Ruan[4], Da Huang[4], Xiaoqun Yang[5], Chaofu Wang[5], Peng Zhang[6], Aki Manninen[1], Liang Wang[3], Gong-Hong Wei[1,6*]

[1] Disease Networks Research Unit, Faculty of Biochemistry and Molecular Medicine & Biocenter Oulu, University of Oulu, Oulu, Finland.

[2] Division of Urology, Department of Surgery, Li Ka Shing Faculty of Medicine, the University of Hong Kong, Hong Kong.

[3] Department of Tumour Biology, H. Lee Moffitt Cancer Center and Research Institute, Tampa, FL, USA.

[4] Department of Urology, Ruijin Hospital, Shanghai Jiaotong University School of Medicine, Shanghai, China.

[5] Department of Pathology, Ruijin Hospital, Shanghai Jiaotong University School of Medicine, Shanghai, China.

[6] Fudan University Shanghai Cancer Center & MOE Key Laboratory of Metabolism and Molecular Medicine and Department of Biochemistry and Molecular Biology of School of Basic Medical Sciences, Shanghai Medical College of Fudan University, Shanghai, China.

# These authors contributed equally.

* Correspondence: gonghong_wei@fudan.edu.cn

This supplementary information file includes the following items:

Supplementary Figures 1-10

Supplementary Tables 1-15

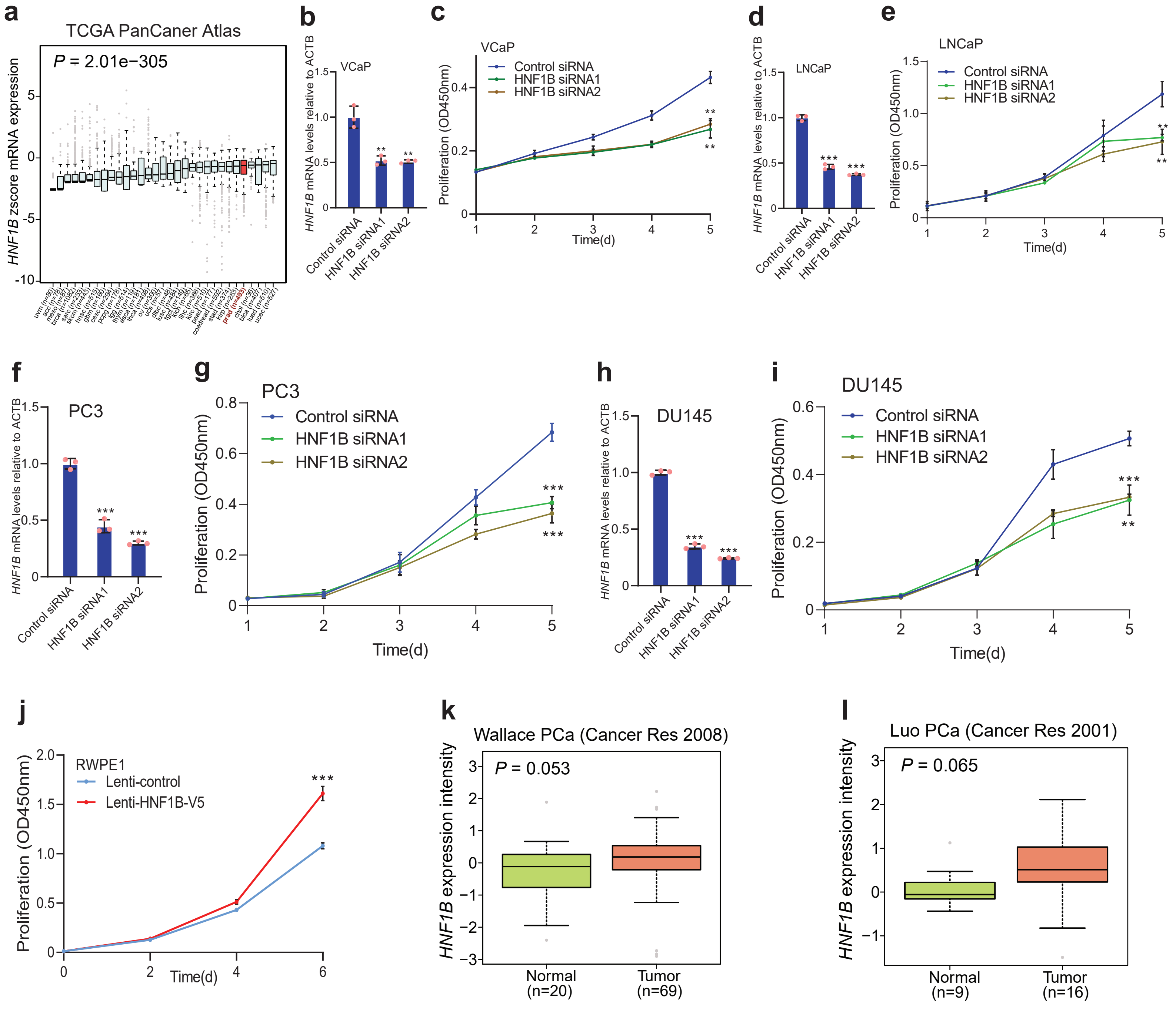

a
TCGA PanCaner Atlas
P = 2.01e−305
HNF1B zscore mRNA expression
b
VCaP
HNF1B mRNA levels relative to ACTB
Control siRNA
HNF1B siRNA1
HNF1B siRNA2
c
VCaP
Control siRNA
HNF1B siRNA1
HNF1B siRNA2
Proliferation (OD450nm)
Time(d)
d
LNCaP
HNF1B mRNA levels relative to ACTB
e
LNCaP
Control siRNA
HNF1B siRNA1
HNF1B siRNA2
Proliferation (OD450nm)
Time(d)
f
PC3
HNF1B mRNA levels relative to ACTB
g
PC3
Control siRNA
HNF1B siRNA1
HNF1B siRNA2
Proliferation (OD450nm)
Time(d)
h
DU145
HNF1B mRNA levels relative to ACTB
i
DU145
Control siRNA
HNF1B siRNA1
HNF1B siRNA2
Proliferation (OD450nm)
Time(d)
j
RWPE1
Lenti-control
Lenti-HNF1B-V5
Proliferation (OD450nm)
Time(d)
k
Wallace PCa (Cancer Res 2008)
P = 0.053
HNF1B expression intensity
Normal (n=20)
Tumor (n=69)
l
Luo PCa (Cancer Res 2001)
P = 0.065
HNF1B expression intensity
Normal (n=9)
Tumor (n=16)

Supplementary Fig. 1: Association of HNF1B with PCa.

a, Expression profiles of HNF1B across 31 cancer types using The Cancer Genome Atlas (TCGA) RNA-seq data (*n*=10,967). *P* value was evaluated by Kruskal-Wallis test. **b-i,** HNF1B knockdown in VCaP (**b**), LNCaP (**d**), PC3 (**f**) and DU145 (**h**) diminish cell proliferation of VCaP (**c**), LNCaP (**e**), PC3 (**g**) and DU145 (**i**) measured by XTT colorimetric assays. (**b-i)** *n*=3 samples;(**b**) *P* values based on the order of appearance: 0,003, 0,002; (**c**) *P* values HNF1B siRNA1: 1,2E-03, HNF1B siRNA2: 1,4E-03; (**d**) *P* values based on the order of appearance: 2,5E-05, 5,5E-06;(**e**) *P* values HNF1B siRNA1: 4,6E-03, HNF1B siRNA2: 9,9E-03; (**f**) *P* values based on the order of appearance: 2E-04, 2E-05;(**g**) *P* values HNF1B siRNA1: 3,6E-04, HNF1B siRNA2: 4,2E-04; (**h**) *P* values based on the order of appearance: 2,7E-06, 3,8E-07;(**i**) *P* values HNF1B siRNA1: 3,1E-03, HNF1B siRNA2: 2,2E-04. **j,** Ectopic expression of *HNF1B* promotes RWPE1 cell proliferation measured by XTT colorimetric assays. *n*=2 samples; *P* values lenti-HNF1B-V5: 1,04E-05. **k-l,** *HNF1B* expression level is elevated in PCa tumours compared to normal prostate tissues in the Wallace (*n*=89) and Luo (*n*=25) cohorts. *P* values were evaluated by the two-sided Mann-Whitney U test. In **a** and **k-l**, the interquartile range (IQR) is depicted by the box with the median represented by the center line. Whiskers maximally extend to 1.5× IQR (with outliers shown). In **b-j**, *n*=3 technical replicates, error bars, mean +/- SD, * $P < 0.05$, ** $P < 0.01$, *** $P < 0.001$, *P* values were assessed using two-tailed Student's t tests. Source data are provided in Source Data file.

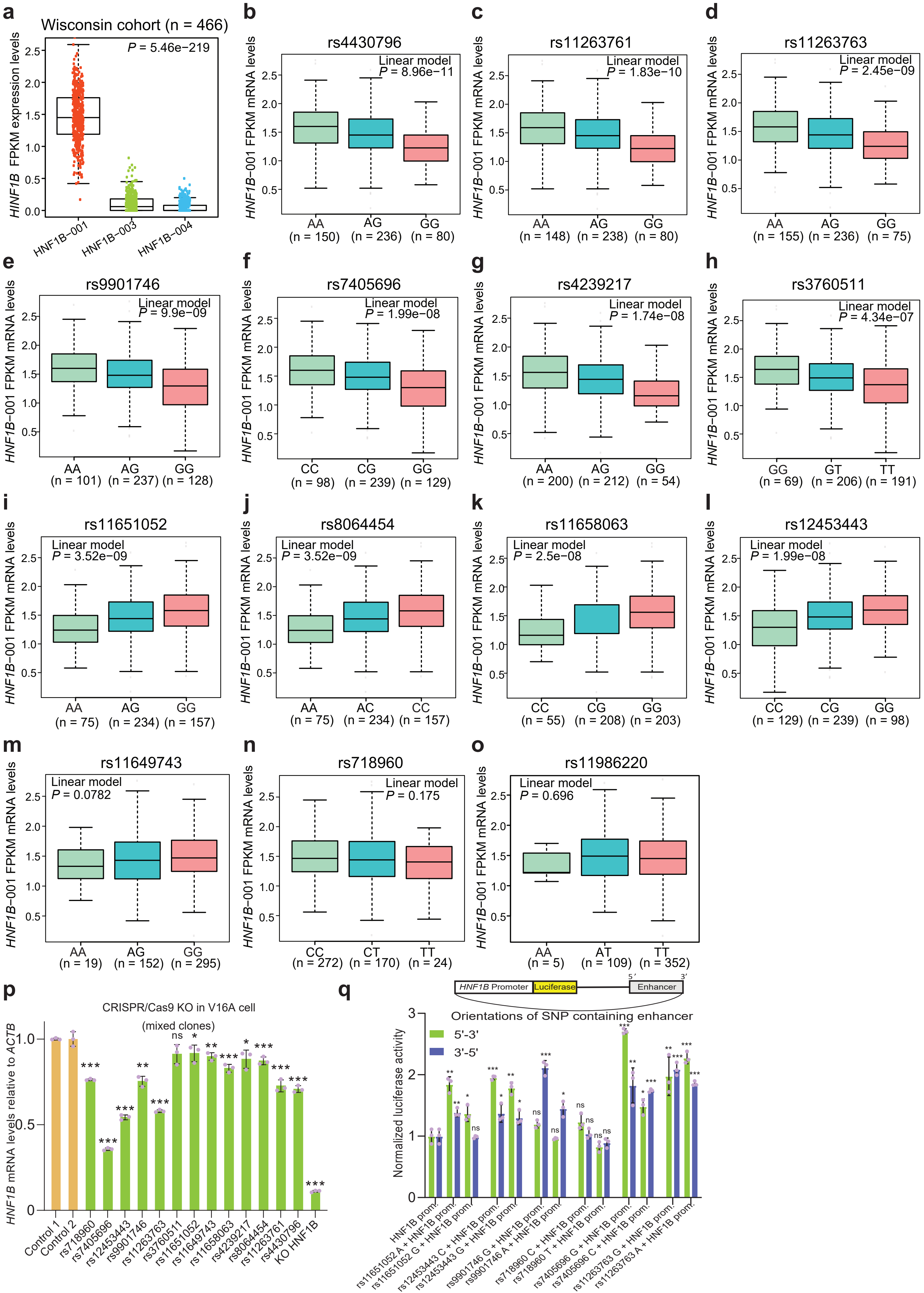

a
Wisconsin cohort (n = 466)
P = 5.46e−219
HNF1B FPKM expression levels
HNF1B−001
HNF1B−003
HNF1B−004
b
rs4430796
Linear model
P = 8.96e−11
AA (n = 150)
AG (n = 236)
GG (n = 80)
c
rs11263761
Linear model
P = 1.83e−10
AA (n = 148)
AG (n = 238)
GG (n = 80)
d
rs11263763
Linear model
P = 2.45e−09
AA (n = 155)
AG (n = 236)
GG (n = 75)
e
rs9901746
Linear model
P = 9.9e−09
AA (n = 101)
AG (n = 237)
GG (n = 128)
f
rs7405696
Linear model
P = 1.99e−08
CC (n = 98)
CG (n = 239)
GG (n = 129)
g
rs4239217
Linear model
P = 1.74e−08
AA (n = 200)
AG (n = 212)
GG (n = 54)
h
rs3760511
Linear model
P = 4.34e−07
GG (n = 69)
GT (n = 206)
TT (n = 191)
i
rs11651052
Linear model
P = 3.52e−09
AA (n = 75)
AG (n = 234)
GG (n = 157)
j
rs8064454
Linear model
P = 3.52e−09
AA (n = 75)
AC (n = 234)
CC (n = 157)
k
rs11658063
Linear model
P = 2.5e−08
CC (n = 55)
CG (n = 208)
GG (n = 203)
l
rs12453443
Linear model
P = 1.99e−08
CC (n = 129)
CG (n = 239)
GG (n = 98)
m
rs11649743
Linear model
P = 0.0782
AA (n = 19)
AG (n = 152)
GG (n = 295)
n
rs718960
Linear model
P = 0.175
CC (n = 272)
CT (n = 170)
TT (n = 24)
o
rs11986220
Linear model
P = 0.696
AA (n = 5)
AT (n = 109)
TT (n = 352)
HNF1B−001 FPKM mRNA levels
p
CRISPR/Cas9 KO in V16A cell
(mixed clones)
HNF1B mRNA levels relative to ACTB
Control 1
Control 2
rs718960
rs7405696
rs12453443
rs9901746
rs11263763
rs3760511
rs11651052
rs11649743
rs11658063
rs4239217
rs8064454
rs11263761
rs4430796
KO HNF1B
q
HNF1B Promoter
Luciferase
Enhancer
Orientations of SNP containing enhancer
5′-3′
3′-5′
Normalized luciferase activity
HNF1B prom.
rs11651052 A + HNF1B prom.
rs11651052 G + HNF1B prom.
rs12453443 C + HNF1B prom.
rs12453443 G + HNF1B prom.
rs9901746 G + HNF1B prom.
rs9901746 A + HNF1B prom.
rs718960 C + HNF1B prom.
rs718960 T + HNF1B prom.
rs7405696 G + HNF1B prom.
rs7405696 C + HNF1B prom.
rs11263763 G + HNF1B prom.
rs11263763 A + HNF1B prom.

Supplementary Fig. 2: An eQTL analysis of the 17q12 locus variants in association with HNF1B expression.

a, HNF1B isoform 1 is dominantly expressed in normal prostate specimens in the Wiscousin cohort (n=466). P value was evaluated by Kruskal-Wallis Test. **b-o,** 11 of the 13 highly associated SNPs were found to have significant eQTL associations with HNF1B isoform 1 in the Wiscousin cohort (n=466). P values were assessed by the linear regression and adjusted by the false discovery rate. **p,** RT-qPCR analysis to determine the *HNF1B* mRNA expression levels in V16A cells with partial knockout for each individual SNP region or dampened *HNF1B* via CRISPR/Cas9 genome editing technology. n=16 samples; P values based on the order of appearance: 7E-07, 3E-08, 9E-07, 1E-04, 2E-07, 0,053, 0,041, 2E-03, 2E-04, 0,019, 7E-04, 2E-04, 2E-05, 4E-09. **q,** Enhancer reporter assays on six SNP regions located in *HNF1B* locus with different alleles and in both orientations (5'-3', 3'-5') cloned along with *HNF1B* promoter in LNCaP. Prom: Promoter. n=25 samples; P values based on the order of appearance: 1E-03, 8,3E-03, 0,022, 0,86, 3E-04, 0,034, 1,4E-03, 0,048, 0,097, 4,8E-04, 0,65, 0,016, 0,105, 0,71, 0,15, 0,32, 3,9E-05, 3,1E-03, 0,012, 9,4E-04, 8E-03, 4,2E-04, 2E-04, 5,2E-04. In **a-o**, the interquartile range (IQR) is depicted by the box with the median represented by the center line. Whiskers maximally extend to 1.5× IQR (with outliers shown). In **p**, **q**, n=3 technical replicates, error bars, mean +/- SD, * $P < 0.05$, ** $P < 0.01$, *** $P < 0.001$, ns: non-significant, P values were assessed using two-tailed Student's t tests. Source data are provided in Source Data file.

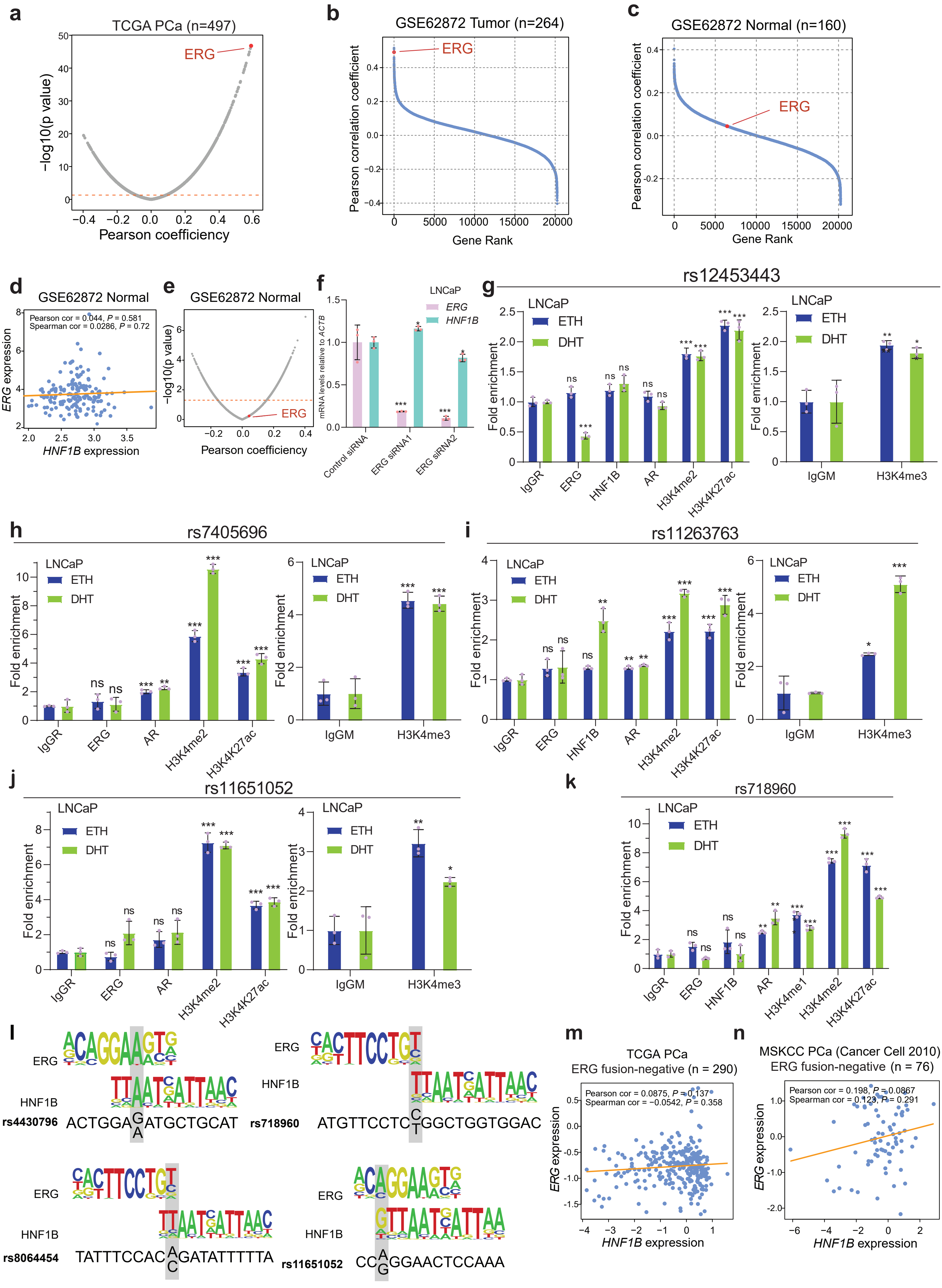

a
TCGA PCa (n=497)
ERG
-log10(p value)
Pearson coefficiency
b
GSE62872 Tumor (n=264)
ERG
Pearson correlation coefficient
Gene Rank
c
GSE62872 Normal (n=160)
ERG
Pearson correlation coefficient
Gene Rank
d
GSE62872 Normal
Pearson cor = 0.044, P = 0.581
Spearman cor = 0.0286, P = 0.72
ERG expression
HNF1B expression
e
GSE62872 Normal
ERG
-log10(p value)
Pearson coefficiency
f
LNCaP
ERG
HNF1B
mRNA levels relative to ACTB
Control siRNA
ERG siRNA1
ERG siRNA2
g
rs12453443
LNCaP
ETH
DHT
Fold enrichment
IgGR
ERG
HNF1B
AR
H3K4me2
H3K4K27ac
IgGM
H3K4me3
h
rs7405696
i
rs11263763
j
rs11651052
k
rs718960
H3K4me1
l
ERG
HNF1B
rs4430796
ACTGGA G/A ATGCTGCAT
rs718960
ATGTTCCTC C/T GGCTGGTGGAC
rs8064454
TATTTCCAC A/C GATATTTTTA
rs11651052
CC A/G GGAACTCCAAA
m
TCGA PCa
ERG fusion-negative (n = 290)
Pearson cor = 0.0875, P = 0.137
Spearman cor = −0.0542, P = 0.358
ERG expression
HNF1B expression
n
MSKCC PCa (Cancer Cell 2010)
ERG fusion-negative (n = 76)
Pearson cor = 0.198, P = 0.0867
Spearman cor = 0.123, P = 0.291
ERG expression
HNF1B expression

Supplementary Fig. 3: ERG is the top-ranking gene showing the highest coexpression correlation with HNF1B in PCa tumours.

a, Pearson correlation displaying *ERG* as the most co-expressed gene with *HNF1B* (*n*=497). **b,** *ERG* is the most co-expressed gene with *HNF1B* in an additional PCa tumour cohort GSE62872 (*n*=264). **c-e,** *ERG* shows no significant co-expression with *HNF1B* in normal prostate glands (*n*=160). In **a**, **e**, *P* values were assessed by the two-sided Pearson's product-moment correlation test. **f,** Decreased mRNA levels of *ERG* have no influence in *HNF1B* mRNA levels in *ERG* non fusion cell line, LNCaP. *n*=3 samples; *P* values based on the order of appearance: 2,3E-03, 0,016, 1,7E-03, 0,016. **g-k,** ChIP-qPCR determination of chromatin enrichment for ERG, HNF1B, AR, H3K4me1, H3K4me2, H3K27ac, and H3K4me3 at rs12453443 (**g**), rs7405696 (**h**), rs11263763 (**i**), rs11651052 (**j**) and rs718960 (**k**) enhancers of the 17q12/*HNF1B* regions in LNCaP cells treated with 100nM DHT and without (ETH-treated). **l,** SNPs rs4430796, rs718960, rs8064454 and rs11651052 reside within HNF1B and ERG DNA-binding motifs. (**g, i**) *n*=16 samples, (**h, j, k**) *n*=14 samples; (**g**) *P* values based on the order of appearance: 0,084, 7,9E-05, 0,05, 0,24, 0,16, 0,45, 3E-04, 1E-04, 3,8E-05, 2,7E-04, 1,2E-03, 0,02; (**h**) *P* values based on the order of appearance: 0,309, 0,748, 2E-04, 8,6E-03, 3E-05, 7E-06, 1E-04, 5E-04, 3E-04, 7E-04; (**i**) *P* values based on the order of appearance: 0,084, 0,26, 0,46, 1,3E-03, 7E-03, 8,6E-03, 6,7E-04, 1,7E-05, 2,4E-04, 2,6E-04, 0,017, 2,2E-05; (**j**) *P* values based on the order of appearance: 0,162, 0,055, 0,052, 0,053, 4,1E-05, 3,5E-06, 3,9E-05, 1E-04, 1,5E-03, 0,025; (**k**) *P* values based on the order of appearance: 0,078, 0,083, 0,17, 0,92, 1,1E-03, 1,1E-03, 1,7E-04, 3,1E-04, 4,8E-06, 3,1E-06, 3,5E-05, 7,8E-06. **m-n,** Expression levels of *ERG* are not significantly correlated with HNF1B in *TMPRSS2-ERG* fusion-negative group in the TCGA (*n*=290) or MSKCC cohort (*n*=76). In **d** and **m-n**, *P* values were assessed by the two-sided Pearson's product-moment correlation and Spearman's rank correlation rho tests. In **f-k**, *n*=3 technical replicates, error bars, mean ± SD, * $P < 0.05$, ** $P < 0.01$, *** $P < 0.001$, ns: non-significant, *P* values were evaluated using two-tailed Student's t-tests. Source data are provided in Source Data file.

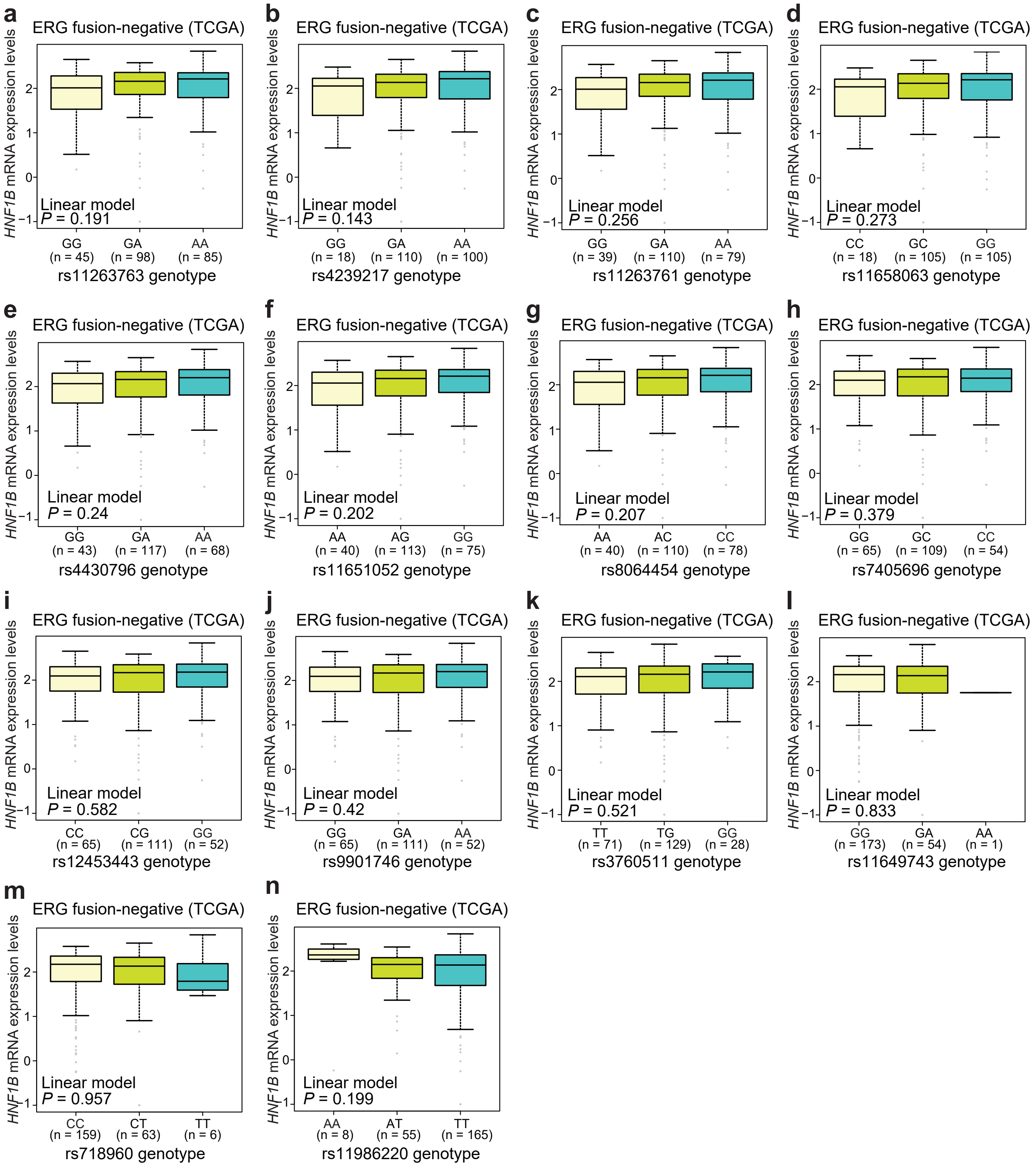
a
ERG fusion-negative (TCGA)
HNF1B mRNA expression levels
Linear model
P = 0.191
GG (n = 45)
GA (n = 98)
AA (n = 85)
rs11263763 genotype
b
ERG fusion-negative (TCGA)
Linear model
P = 0.143
GG (n = 18)
GA (n = 110)
AA (n = 100)
rs4239217 genotype
c
ERG fusion-negative (TCGA)
Linear model
P = 0.256
GG (n = 39)
GA (n = 110)
AA (n = 79)
rs11263761 genotype
d
ERG fusion-negative (TCGA)
Linear model
P = 0.273
CC (n = 18)
GC (n = 105)
GG (n = 105)
rs11658063 genotype
e
ERG fusion-negative (TCGA)
Linear model
P = 0.24
GG (n = 43)
GA (n = 117)
AA (n = 68)
rs4430796 genotype
f
ERG fusion-negative (TCGA)
Linear model
P = 0.202
AA (n = 40)
AG (n = 113)
GG (n = 75)
rs11651052 genotype
g
ERG fusion-negative (TCGA)
Linear model
P = 0.207
AA (n = 40)
AC (n = 110)
CC (n = 78)
rs8064454 genotype
h
ERG fusion-negative (TCGA)
Linear model
P = 0.379
GG (n = 65)
GC (n = 109)
CC (n = 54)
rs7405696 genotype
i
ERG fusion-negative (TCGA)
Linear model
P = 0.582
CC (n = 65)
CG (n = 111)
GG (n = 52)
rs12453443 genotype
j
ERG fusion-negative (TCGA)
Linear model
P = 0.42
GG (n = 65)
GA (n = 111)
AA (n = 52)
rs9901746 genotype
k
ERG fusion-negative (TCGA)
Linear model
P = 0.521
TT (n = 71)
TG (n = 129)
GG (n = 28)
rs3760511 genotype
l
ERG fusion-negative (TCGA)
Linear model
P = 0.833
GG (n = 173)
GA (n = 54)
AA (n = 1)
rs11649743 genotype
m
ERG fusion-negative (TCGA)
Linear model
P = 0.957
CC (n = 159)
CT (n = 63)
TT (n = 6)
rs718960 genotype
n
ERG fusion-negative (TCGA)
Linear model
P = 0.199
AA (n = 8)
AT (n = 55)
TT (n = 165)
rs11986220 genotype

Supplementary Fig. 4: eQTL association with HNF1B in TMPRSS2-ERG fusion-negative group of PCa patients.

a-n, None of the 13 highly associated SNPs in the 17q12/*HNF1B* locus was found to have significant eQTL association with HNF1B in *TMPRSS2-ERG* fusion-negative PCa patients of TCGA cohort (*n*=228). Genotype to phenotype correlations were evaluated with linear regression. *P* values are adjusted by false discovery rate. The interquartile range (IQR) is depicted by the box with the median represented by the center line. Whiskers maximally extend to 1.5× IQR (with outliers shown). Source data are provided in Source Data file.

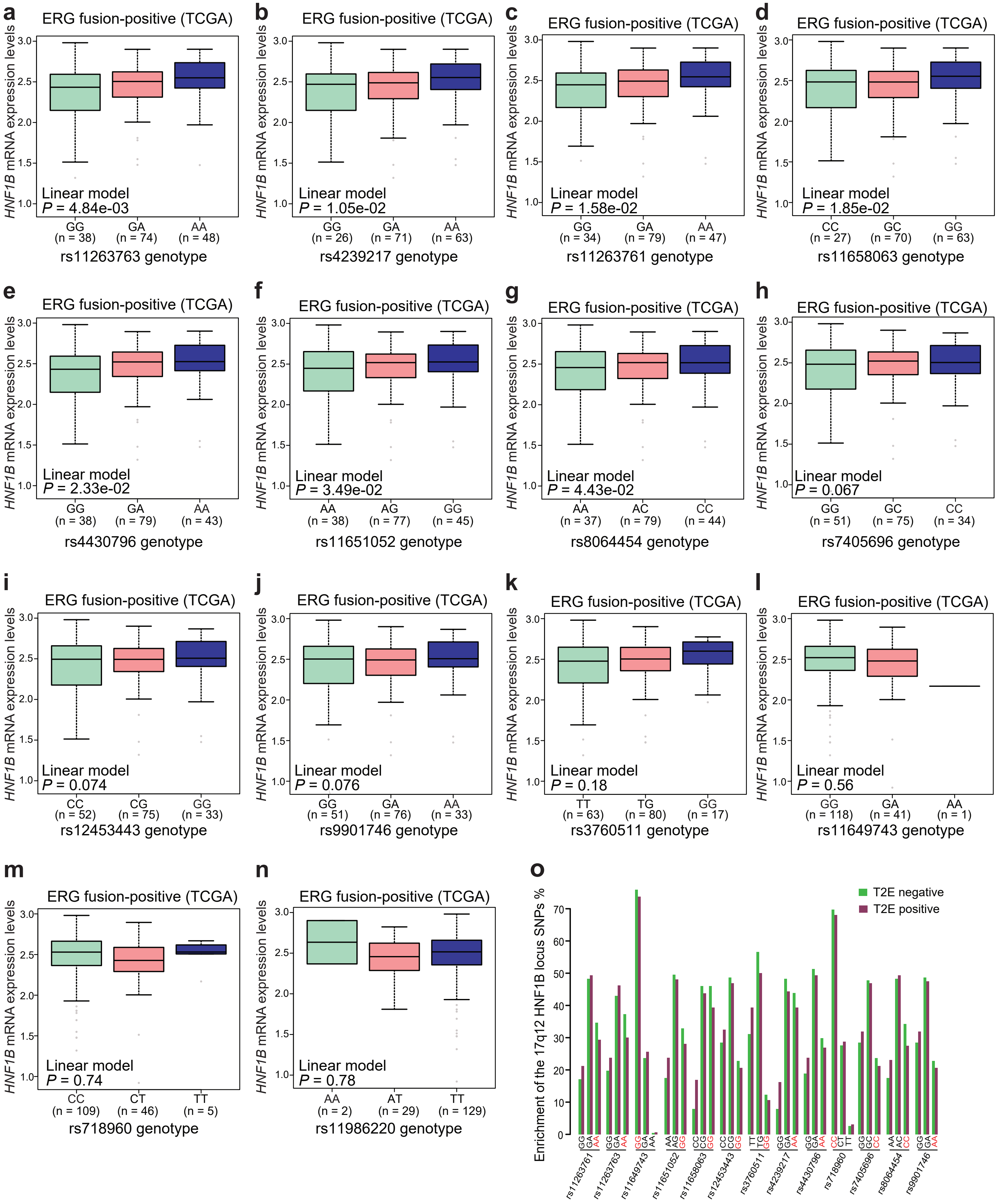

a
ERG fusion-positive (TCGA)
HNF1B mRNA expression levels
Linear model
P = 4.84e-03
GG (n = 38)
GA (n = 74)
AA (n = 48)
rs11263763 genotype
b
ERG fusion-positive (TCGA)
Linear model
P = 1.05e-02
GG (n = 26)
GA (n = 71)
AA (n = 63)
rs4239217 genotype
c
ERG fusion-positive (TCGA)
Linear model
P = 1.58e-02
GG (n = 34)
GA (n = 79)
AA (n = 47)
rs11263761 genotype
d
ERG fusion-positive (TCGA)
Linear model
P = 1.85e-02
CC (n = 27)
GC (n = 70)
GG (n = 63)
rs11658063 genotype
e
ERG fusion-positive (TCGA)
Linear model
P = 2.33e-02
GG (n = 38)
GA (n = 79)
AA (n = 43)
rs4430796 genotype
f
ERG fusion-positive (TCGA)
Linear model
P = 3.49e-02
AA (n = 38)
AG (n = 77)
GG (n = 45)
rs11651052 genotype
g
ERG fusion-positive (TCGA)
Linear model
P = 4.43e-02
AA (n = 37)
AC (n = 79)
CC (n = 44)
rs8064454 genotype
h
ERG fusion-positive (TCGA)
Linear model
P = 0.067
GG (n = 51)
GC (n = 75)
CC (n = 34)
rs7405696 genotype
i
ERG fusion-positive (TCGA)
Linear model
P = 0.074
CC (n = 52)
CG (n = 75)
GG (n = 33)
rs12453443 genotype
j
ERG fusion-positive (TCGA)
Linear model
P = 0.076
GG (n = 51)
GA (n = 76)
AA (n = 33)
rs9901746 genotype
k
ERG fusion-positive (TCGA)
Linear model
P = 0.18
TT (n = 63)
TG (n = 80)
GG (n = 17)
rs3760511 genotype
l
ERG fusion-positive (TCGA)
Linear model
P = 0.56
GG (n = 118)
GA (n = 41)
AA (n = 1)
rs11649743 genotype
m
ERG fusion-positive (TCGA)
Linear model
P = 0.74
CC (n = 109)
CT (n = 46)
TT (n = 5)
rs718960 genotype
n
ERG fusion-positive (TCGA)
Linear model
P = 0.78
AA (n = 2)
AT (n = 29)
TT (n = 129)
rs11986220 genotype
o
T2E negative
T2E positive
Enrichment of the 17q12 HNF1B locus SNPs %
rs11263761
rs11263763
rs11649743
rs11651052
rs11658063
rs12453443
rs3760511
rs4239217
rs4430796
rs718960
rs7405696
rs8064454
rs9901746

Supplementary Fig. 5: eQTL association with HNF1B in TMPRSS2-ERG fusion-positive group of PCa patients.

a-n, Four of the 13 highly associated SNPs at the 17q12/*HNF1B* locus were found to have significant eQTL association with *HNF1B* expression levels in PCa patient group with *TMPRSS2-ERG* fusion (n=160). Genotype to phenotype correlations were evaluated with linear regression. P values are adjusted by false discovery rate. The interquartile range (IQR) is depicted by the box with the median represented by the center line. Whiskers maximally extend to 1.5× IQR (with outliers shown). **o,** Enrichment of the 17q12 *HNF1B* locus SNPs in *TMPRSS2-ERG* fusion-positive and -negative tumours in the TCGA cohort (n=388). Source data are provided in Source Data file.

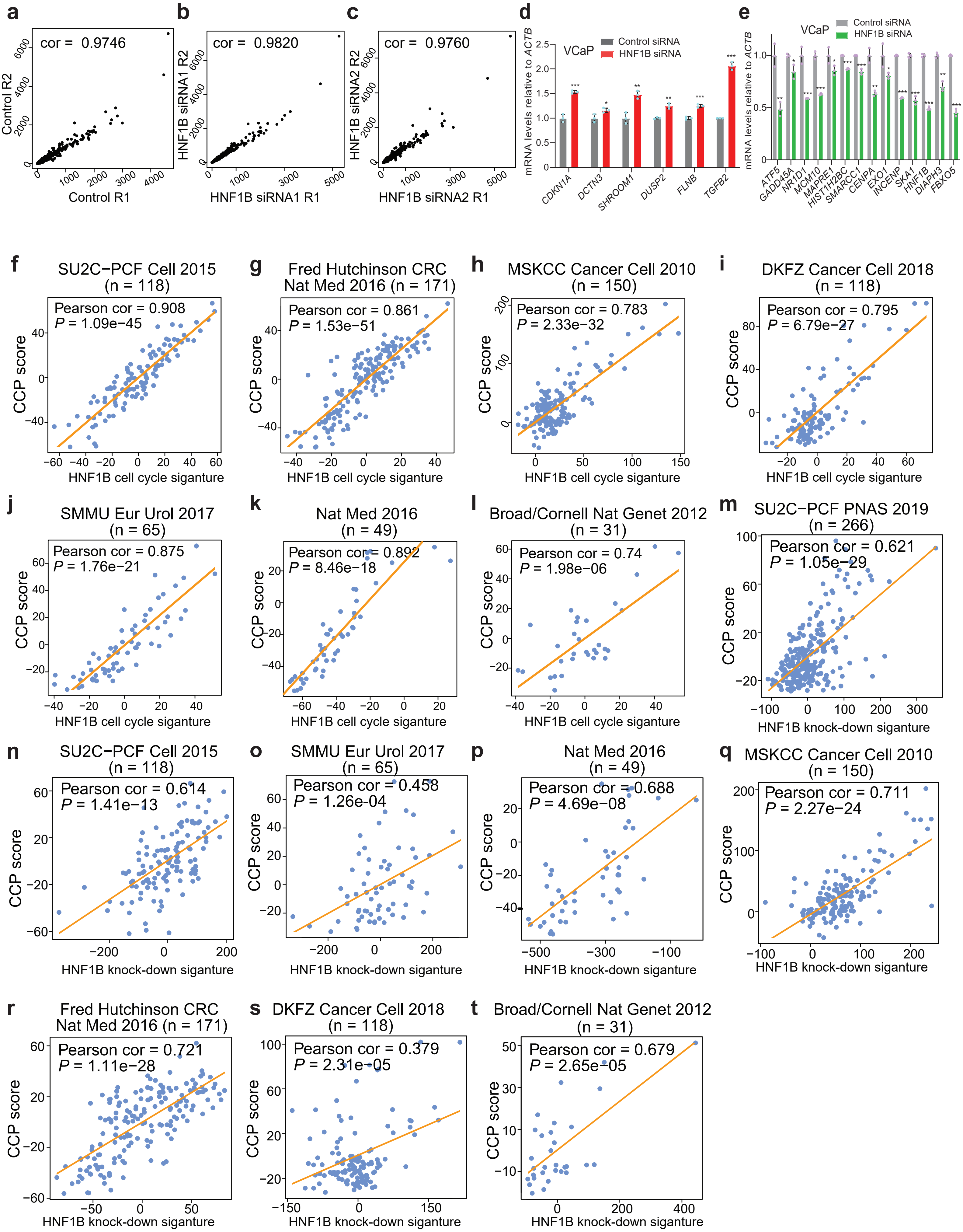

a
cor = 0.9746
Control R2
Control R1
b
cor = 0.9820
HNF1B siRNA1 R2
HNF1B siRNA1 R1
c
cor = 0.9760
HNF1B siRNA2 R2
HNF1B siRNA2 R1
d
VCaP
Control siRNA
HNF1B siRNA
mRNA levels relative to ACTB
CDKN1A
DCTN3
SHROOM1
DUSP2
FLNB
TGFB2
e
VCaP
Control siRNA
HNF1B siRNA
mRNA levels relative to ACTB
ATF5
GADD45A
NR1D1
MCM10
MAPRE1
HIST1H2BC
SMARCC1
CENPA
EXO1
INCENP
SKA1
HNF1B
DIAPH3
FBXO5
f
SU2C−PCF Cell 2015 (n = 118)
Pearson cor = 0.908
P = 1.09e−45
CCP score
HNF1B cell cycle siganture
g
Fred Hutchinson CRC Nat Med 2016 (n = 171)
Pearson cor = 0.861
P = 1.53e−51
h
MSKCC Cancer Cell 2010 (n = 150)
Pearson cor = 0.783
P = 2.33e−32
i
DKFZ Cancer Cell 2018 (n = 118)
Pearson cor = 0.795
P = 6.79e−27
j
SMMU Eur Urol 2017 (n = 65)
Pearson cor = 0.875
P = 1.76e−21
k
Nat Med 2016 (n = 49)
Pearson cor = 0.892
P = 8.46e−18
l
Broad/Cornell Nat Genet 2012 (n = 31)
Pearson cor = 0.74
P = 1.98e−06
m
SU2C−PCF PNAS 2019 (n = 266)
Pearson cor = 0.621
P = 1.05e−29
HNF1B knock-down siganture
n
SU2C−PCF Cell 2015 (n = 118)
Pearson cor = 0.614
P = 1.41e−13
o
SMMU Eur Urol 2017 (n = 65)
Pearson cor = 0.458
P = 1.26e-04
p
Nat Med 2016 (n = 49)
Pearson cor = 0.688
P = 4.69e−08
q
MSKCC Cancer Cell 2010 (n = 150)
Pearson cor = 0.711
P = 2.27e−24
r
Fred Hutchinson CRC Nat Med 2016 (n = 171)
Pearson cor = 0.721
P = 1.11e−28
s
DKFZ Cancer Cell 2018 (n = 118)
Pearson cor = 0.379
P = 2.31e−05
t
Broad/Cornell Nat Genet 2012 (n = 31)
Pearson cor = 0.679
P = 2.65e−05

Supplementary Fig. 6: Experimental validation of HNF1B target genes and exploration of their clinical relevance.

a-c, RPKM expression correlation between two biological replicates of control siRNA, HNF1B siRNA1, or HNF1B siRNA2, respectively. **d-e,** RT-qPCR validation of HNF1B positively or negatively regulated target genes revealed by RNA-seq profiling. (**d**, **e**) *n*=2 samples; (**d**) *P* values based on the order of appearance: 4E-04, 0,04, 4E-04, 1E-04, 5E-04, 2E-05; (**e**) *P* values based on the order of appearance: 0,002, 0,016, 9E-04, 7E-05, 0,049, 2E-05, 6E-04, 1E-03, 0,042, 2E-07, 7E-05, 2E-06, 6E-04, 1E-05. **f-l,** HNF1B cell-cycle gene signature score indicates significant positive Pearson correlation with the Cell Cycle Progression (CCP) scores across several independent cohorts of PCa patients (**f**, *n*=118), (**g**, *n*=171), (**h**, *n*=150), (**i**, *n*=118), (**j**, *n*=65), (**k**, *n*=49), and (**l**, *n*=31). **m-t,** Scatter plots displaying significant positive linear correlation between HNF1B knock-down signature and CCP scores in eight independent cohorts of PCa patients (**m**, *n*=266), (**n**, *n*=118), (**o**, *n*=65), (**p**, *n*=49), (**q**, *n*=150), (**r**, *n*=171), (**s**, *n*=118), and (**t**, *n*=31). HNF1B knock-down signature score was calculated by the z-score sum of the 339 differentially expressed genes upon HNF1B siRNA knockdown followed by RNA-seq profiling. In **d**, **e**, *n*=3 technical replicates, error bars, mean +/- SD, * $P < 0.05$, ** $P < 0.01$, *** $P < 0.001$, *P* values were assessed using two-tailed Student's t tests. In **f**-**t**, *P* values were assessed by the two-sided Pearson's product-moment correlation test. Source data are provided in Source Data file.

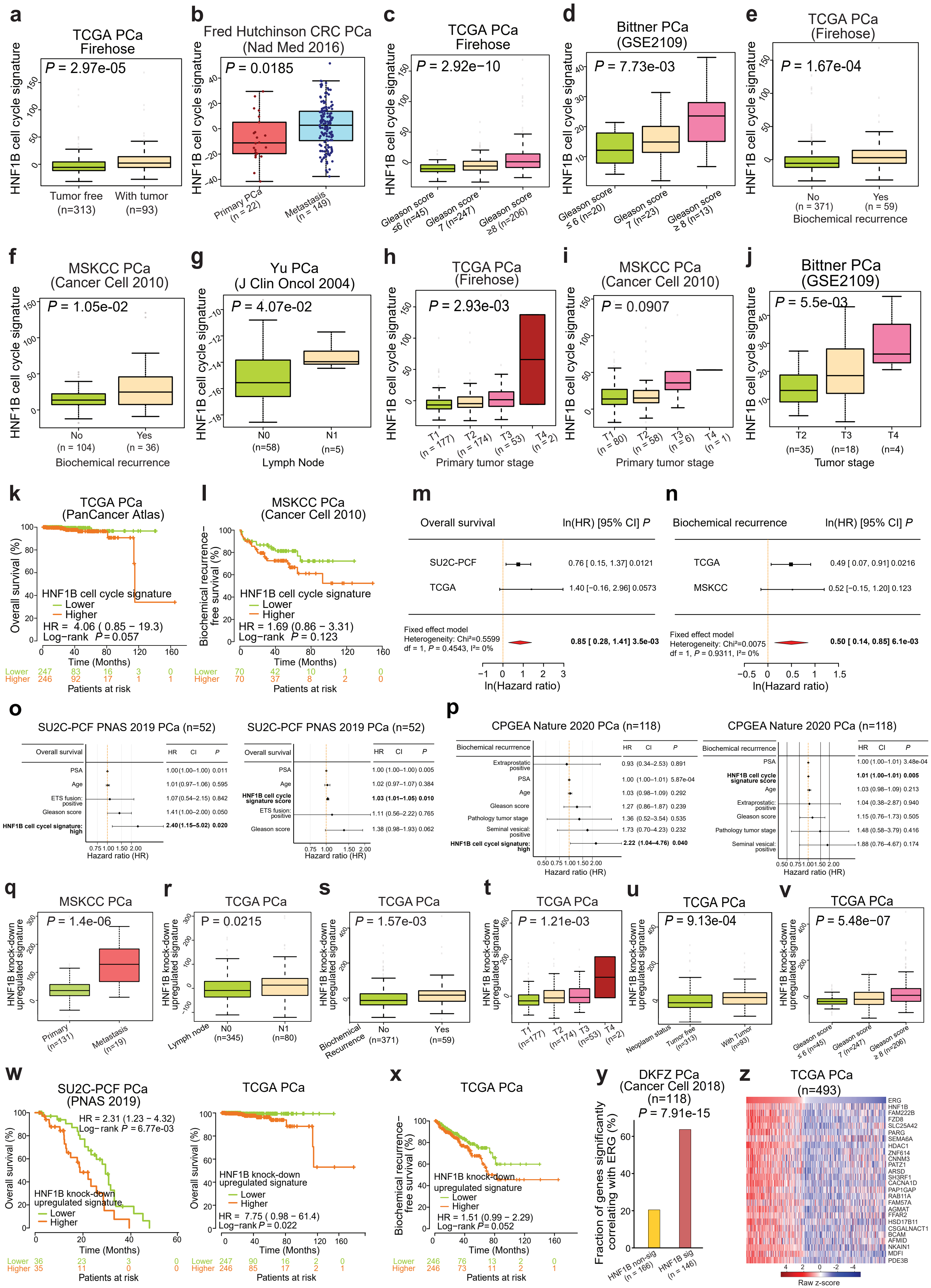
a
TCGA PCa Firehose
P = 2.97e-05
HNF1B cell cycle signature
Tumor free (n=313)
With tumor (n=93)
b
Fred Hutchinson CRC PCa (Nad Med 2016)
P = 0.0185
Primary PCa (n = 22)
Metastasis (n = 149)
c
TCGA PCa Firehose
P = 2.92e−10
Gleason score ≤6 (n=45)
Gleason score 7 (n=247)
Gleason score ≥8 (n=206)
d
Bittner PCa (GSE2109)
P = 7.73e-03
Gleason score ≤ 6 (n=20)
Gleason score 7 (n=23)
Gleason score ≥ 8 (n=13)
e
TCGA PCa (Firehose)
P = 1.67e-04
No (n = 371)
Yes (n = 59)
Biochemical recurrence
f
MSKCC PCa (Cancer Cell 2010)
P = 1.05e-02
No (n = 104)
Yes (n = 36)
Biochemical recurrence
g
Yu PCa (J Clin Oncol 2004)
P = 4.07e-02
N0 (n=58)
N1 (n=5)
Lymph Node
h
TCGA PCa (Firehose)
P = 2.93e-03
T1 (n = 177)
T2 (n = 174)
T3 (n = 53)
T4 (n = 2)
Primary tumor stage
i
MSKCC PCa (Cancer Cell 2010)
P = 0.0907
T1 (n = 80)
T2 (n = 58)
T3 (n = 6)
T4 (n = 1)
Primary tumor stage
j
Bittner PCa (GSE2109)
P = 5.5e-03
T2 (n=35)
T3 (n=18)
T4 (n=4)
Tumor stage
k
TCGA PCa (PanCancer Atlas)
Overall survival (%)
HNF1B cell cycle signature
Lower
Higher
HR = 4.06 (0.85 − 19.3)
Log−rank P = 0.057
Time (Months)
Lower 247 83 16 3 0
Higher 246 92 17 1 1
Patients at risk
l
MSKCC PCa (Cancer Cell 2010)
Biochemical recurrence−free survival (%)
HNF1B cell cycle signature
Lower
Higher
HR = 1.69 (0.86 − 3.31)
Log−rank P = 0.123
Time (Months)
Lower 70 42 10 1 0
Higher 70 37 8 2 0
Patients at risk
m
Overall survival ln(HR) [95% CI] P
SU2C-PCF 0.76 [0.15, 1.37] 0.0121
TCGA 1.40 [−0.16, 2.96] 0.0573
Fixed effect model
Heterogeneity: Chi²=0.5599
df = 1, P = 0.4543, I² = 0%
0.85 [0.28, 1.41] 3.5e-03
ln(Hazard ratio)
n
Biochemical recurrence ln(HR) [95% CI] P
TCGA 0.49 [0.07, 0.91] 0.0216
MSKCC 0.52 [−0.15, 1.20] 0.123
Fixed effect model
Heterogeneity: Chi²=0.0075
df = 1, P = 0.9311, I² = 0%
0.50 [0.14, 0.85] 6.1e-03
ln(Hazard ratio)
o
SU2C-PCF PNAS 2019 PCa (n=52)
Overall survival HR CI P
PSA 1.00 (1.00−1.00) 0.011
Age 1.01 (0.97−1.06) 0.595
ETS fusion: positive 1.07 (0.54−2.15) 0.842
Gleason score 1.41 (1.00−2.00) 0.050
HNF1B cell cycel signature: high 2.40 (1.15−5.02) 0.020
Hazard ratio (HR)
SU2C-PCF PNAS 2019 PCa (n=52)
Overall survival HR CI P
PSA 1.00 (1.00−1.00) 0.005
Age 1.02 (0.97−1.07) 0.384
HNF1B cell cycle signature score 1.03 (1.01−1.05) 0.010
ETS fusion: positive 1.11 (0.56−2.22) 0.765
Gleason score 1.38 (0.98−1.93) 0.062
Hazard ratio (HR)
p
CPGEA Nature 2020 PCa (n=118)
Biochemical recurrence HR CI P
Extraprostatic: positive 0.93 (0.34−2.53) 0.891
PSA 1.00 (1.00−1.01) 5.87e-04
Age 1.03 (0.98−1.09) 0.292
Gleason score 1.27 (0.86−1.87) 0.239
Pathology tumor stage 1.36 (0.52−3.54) 0.535
Seminal vesical: positive 1.73 (0.70−4.23) 0.232
HNF1B cell cycel signature: high 2.22 (1.04−4.76) 0.040
Hazard ratio (HR)
CPGEA Nature 2020 PCa (n=118)
Biochemical recurrence HR CI P
PSA 1.00 (1.00−1.01) 3.48e-04
HNF1B cell cycle signature score 1.01 (1.00−1.01) 0.005
Age 1.03 (0.98−1.09) 0.213
Extraprostatic: positive 1.04 (0.38−2.87) 0.940
Gleason score 1.15 (0.76−1.73) 0.505
Pathology tumor stage 1.48 (0.58−3.79) 0.416
Seminal vesical: positive 1.88 (0.76−4.67) 0.174
Hazard ratio (HR)
q
MSKCC PCa
P = 1.4e−06
HNF1B knock-down upregulated signature
Primary (n=131)
Metastasis (n=19)
r
TCGA PCa
P = 0.0215
Lymph node N0 (n=345) N1 (n=80)
s
TCGA PCa
P = 1.57e−03
Biochemical Recurrence No (n=371) Yes (n=59)
t
TCGA PCa
P = 1.21e−03
T1 (n=177) T2 (n=174) T3 (n=53) T4 (n=2)
u
TCGA PCa
P = 9.13e−04
Neoplasm status Tumor free (n=313) With Tumor (n=93)
v
TCGA PCa
P = 5.48e−07
Gleason score ≤ 6 (n=45)
Gleason score 7 (n=247)
Gleason score ≥ 8 (n=206)
w
SU2C-PCF PCa (PNAS 2019)
HR = 2.31 (1.23 − 4.32)
Log−rank P = 6.77e−03
Overall survival (%)
HNF1B knock-down upregulated signature
Lower
Higher
Time (Months)
Lower 36 23 3 0
Higher 35 11 0 0
Patients at risk
TCGA PCa
Overall survival (%)
HNF1B knock-down upregulated signature
Lower
Higher
HR = 7.75 (0.98 − 61.4)
Log−rank P = 0.022
Time (Months)
Lower 247 90 16 2 0
Higher 246 85 17 2 1
Patients at risk
x
TCGA PCa
Biochemical recurrence−free survival (%)
HNF1B knock-down upregulated signature
Lower
Higher
HR = 1.51 (0.99 − 2.29)
Log−rank P = 0.052
Time (Months)
Lower 246 76 13 2 0
Higher 246 73 11 2 1
Patients at risk
y
DKFZ PCa (Cancer Cell 2018) (n=118)
P = 7.91e-15
Fraction of genes significantly correlating with ERG (%)
HNF1B non-sig (n = 166)
HNF1B sig (n = 146)
z
TCGA PCa (n=493)
ERG
HNF1B
FAM222B
FZD8
SLC25A42
PARG
SEMA6A
HDAC1
ZNF614
CNNM3
PATZ1
ARSD
SH3RF1
CACNA1D
PAP1GAP
RAB11A
FAM57A
AGMAT
FFAR2
HSD17B11
CSGALNACT1
BCAM
AFMID
NKAIN1
MDFI
PDE3B
Raw z-score

Supplementary Fig. 7: Characterization of clinical relevance for HNF1B target gene signatures.

a-j, The HNF1B cell cycle signature was upregulated upon PCa tumour development and progression (**a**, *n*=406), (**b**, *n*=171), (**c**, *n*=498), (**d**, *n*=56), (**e**, *n*=430), (**f**, *n*=140), (**g**, *n*=63), (**h**, *n*=406), (**i**, *n*=145), and (**j**, *n*=57). *P* values were calculated using the two-sided Mann-Whitney U test or Kruskal-Wallis test. **k, l,** Kaplan-Meier plots showing the overall survival (**k**, *n*=493) and biochemical relapse (**l**, *n*=140) of PCa patients. *P* values were calculated using log-rank test. **m, n,** Forest plots displaying the meta-analysis of the hazard ratio estimates for overall survival in SU2C-PCF (*n*=71) and TCGA (*n*=493) cohorts (**m**), and biochemical recurrence in TCGA (*n*=492) and MSKCC (*n*=140) cohorts (**n**). The horizontal error bars represent the 95% CI with the measure of centre as HR. The HR and 95% CI were presented in the form of natural logarithm (ln). *P* values were calculated by the two-way Fixed-Effects Model. **o, p,** Multivariate analysis of the risk for patient overall survival (**o**, *n*=52) and biochemical recurrence (**p**, *n*=118) in PCa tumours with several clinical variables and the categorical or continuous value of the HNF1B cell cycle signature. *P* values were evaluated by the Cox's proportional hazards regression. Forest plot displays the measurement of HR with horizontal error bars representing the 95% CI. **q-v,** Correlations between HNF1B knock-down upregulated signature and PCa severity (**q**, *n*=150), (**r**, *n*=425), (**s**, *n*=430), (**t**, *n*=406), (**u**, *n*=406), and (**v**, *n*=498). *P* values were calculated using the two-sided Mann–Whitney U test or Kruskal-Wallis test. **w, x,** Kaplan-Meier plots showing rates of overall survival (**w**, *n*=71, 493) and biochemical relapse (**x**, *n*=492) of PCa patients. *P* values were calculated using log-rank test. **y,** Differentially expressed genes that are significantly co-expressed with *HNF1B* indicate increased likelihood to be greatly co-expressed with TMPRSS2-ERG (*n*=118). *P* value was evaluated by the two-sided Fisher's exact test. **z,** A 25-gene co-expression signature with *HNF1B* displayed a similar expression pattern with *ERG* (*n*=493). In **a-j** and **q-v**, the interquartile range (IQR) is depicted by the box with the median represented by the center line. Whiskers maximally extend to 1.5× IQR (with outliers shown). HR, hazard ratio; CI, confidence interval. Source data are provided in Source Data file.

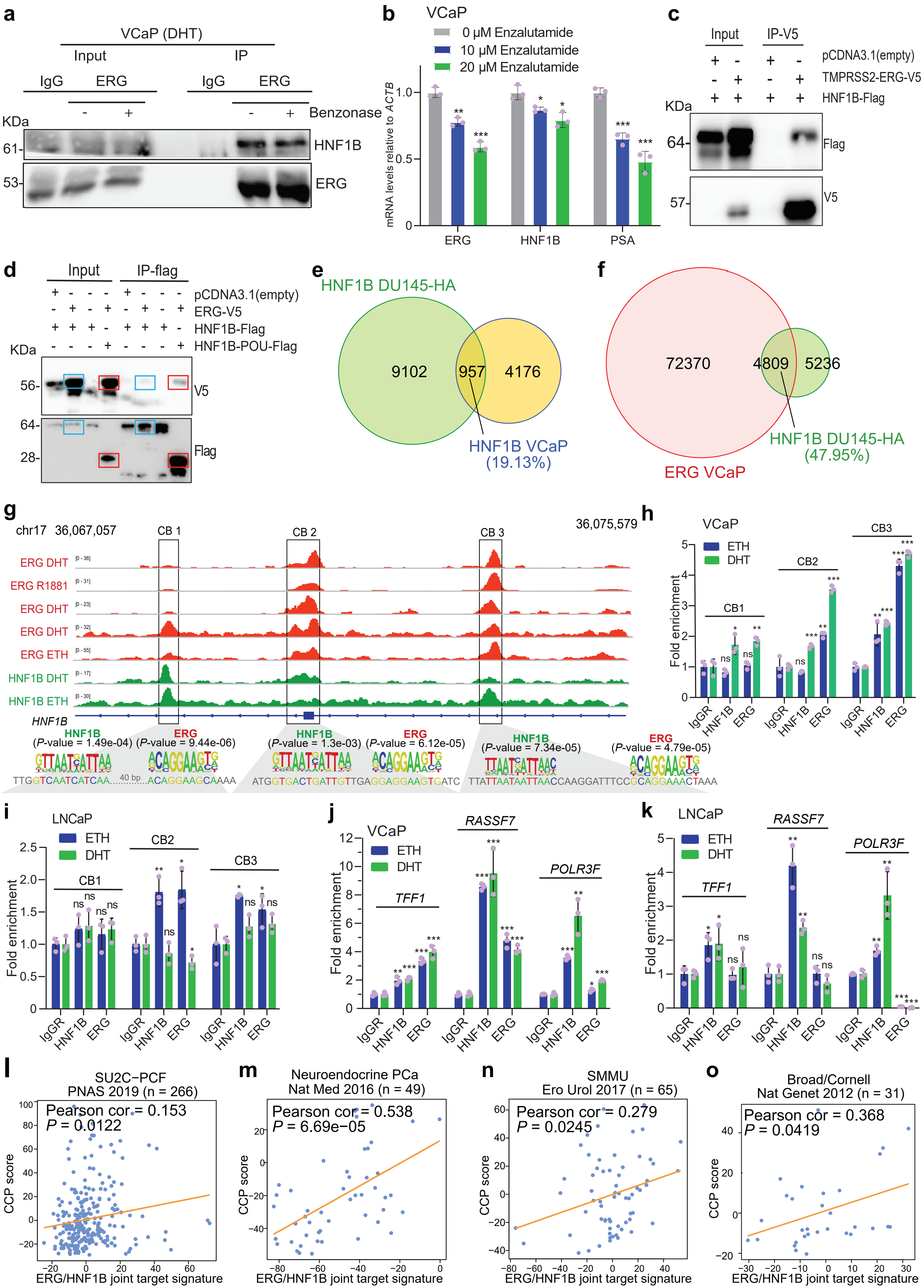

a
VCaP (DHT)
Input
IP
IgG
ERG
Benzonase
KDa
61-
53-
HNF1B
ERG
b
VCaP
0 μM Enzalutamide
10 μM Enzalutamide
20 μM Enzalutamide
mRNA levels relative to ACTB
ERG
HNF1B
PSA
c
Input
IP-V5
pCDNA3.1(empty)
TMPRSS2-ERG-V5
HNF1B-Flag
KDa
64-
57-
Flag
V5
d
Input
IP-flag
pCDNA3.1(empty)
ERG-V5
HNF1B-Flag
HNF1B-POU-Flag
KDa
56-
64-
28-
V5
Flag
e
HNF1B DU145-HA
9102
957
4176
HNF1B VCaP
(19.13%)
f
72370
4809
5236
HNF1B DU145-HA
(47.95%)
ERG VCaP
g
chr17 36,067,057
36,075,579
CB 1
CB 2
CB 3
ERG DHT
ERG R1881
ERG DHT
ERG DHT
ERG ETH
HNF1B DHT
HNF1B ETH
HNF1B
HNF1B (P-value = 1.49e-04)
ERG (P-value = 9.44e-06)
HNF1B (P-value = 1.3e-03)
ERG (P-value = 6.12e-05)
HNF1B (P-value = 7.34e-05)
ERG (P-value = 4.79e-05)
TTGGTCAATCATCAA.......40 bp.....ACAGGAAGCAAAA
ATGGTGACTGATTGTTGAGGAGGAAGTGATC
TTATTAATAATTAACCAAGGATTTCCGCAGGAAACTAAA
h
VCaP
ETH
DHT
CB1
CB2
CB3
Fold enrichment
IgGR
HNF1B
ERG
i
LNCaP
ETH
DHT
CB1
CB2
CB3
Fold enrichment
j
VCaP
ETH
DHT
TFF1
RASSF7
POLR3F
Fold enrichment
k
LNCaP
ETH
DHT
TFF1
RASSF7
POLR3F
Fold enrichment
l
SU2C−PCF
PNAS 2019 (n = 266)
Pearson cor = 0.153
P = 0.0122
CCP score
ERG/HNF1B joint target signature
m
Neuroendocrine PCa
Nat Med 2016 (n = 49)
Pearson cor = 0.538
P = 6.69e−05
CCP score
ERG/HNF1B joint target signature
n
SMMU
Ero Urol 2017 (n = 65)
Pearson cor = 0.279
P = 0.0245
CCP score
ERG/HNF1B joint target signature
o
Broad/Cornell
Nat Genet 2012 (n = 31)
Pearson cor = 0.368
P = 0.0419
CCP score
ERG/HNF1B joint target signature

Supplementary Fig. 8: Physical interaction of HNF1B and ERG, and their direct target gene signature in the clinical setting.

a, Physical interaction of endogenous expression of HNF1B and ERG protein with benzonase treatment in VCaP treated with 100nM. **b,** RT-qPCR analysis of the mRNA levels of *ERG, HNF1B* and *PSA* with enzalutamide treatment for 72hrs. *n*=3 samples; *P* values based on the order of appearance: 2E-03, 2E-04, 0,018, 0,011, 4E-04, 4E-04. **c,** Exogenous interaction of recombinant HNF1B and TMPRSS2-ERG proteins in 293T cells. **d,** Interaction of recombinant HNF1B protein with exogenous ERG in vitro via the POU domain defined by co-IP with anti-flag antibody in 293T cells. **e,** HNF1B in VCaP cells shares 19.13% of common chromatin binding sites with that of HNF1B in DU145 cells. **f,** 47.95% of HNF1B binding sites in DU145 cells are co-occupied by that of ERG in VCaP cells. **g,** HNF1B and ERG chromatin binding sites of at the *HNF1B* locus. Upper: Common binding regions of HNF1B and ERG. Genomic coordinates in hg19 assembly. Lower: FIMO sequence scan for ERG and HNF1B motifs at common binding regions. **h, i,** ChIP-qPCR validation for chromatin enrichment of ERG and HNF1B at common binding (CB) sites at *HNF1B* locus in VCaP and LNCaP cells treated with 100nM DHT and without (ETH-treated). (**h**,**i**) *n*=6 samples; (**h**) *P* values based on the order of appearance: 0,23, 0,036, 0,71, 7E-03, 0,45, 6E-04, 6E-03, 7E-06, 9E-03, 7E-06, 2E-05, 5E-07; (**i**) *P* values based on the order of appearance: 0,18, 0,14, 0,37, 0,13, 5E-03, 0,31, 0,01, 0,042, 0,011, 0,089, 0,075, 0,047. **j, k,** ChIP-qPCR validation of **Fig. 5g** for chromatin enrichment of ERG and HNF1B at *TFF1*, *RASSF7* and *POLR3F* loci in VCaP and LNCaP cells treated with 100nM DHT and without (ETH-treated). (**j**,**k**) *n*=6 samples; (**j**) *P* values based on the order of appearance: 5E-03, 6E-04, 8E-05, 4E-04, 8E-07, 9E-04, 2E-04, 8E-05, 1E-05, 2E-03, 0,01, 2E-04; (**k**) *P* values based on the order of appearance: 0,03, 0,039, 0,87, 0,57, 1E-03, 1E-03, 0,96, 0,22, 1E-03, 5E-03, 1E-07, 1E-04. **l-o** Pearson correlation tests demonstrate significant positive linear correlation between HNF1B&ERG direct target gene signature scores and Cell Cycle Progression signature scores in four independent PCa cohorts (**l**, *n*=266), (**m**, *n*=49), (**n**, *n*=65), and (**o**, *n*=31). *P* values were assessed by the two-sided Pearson's product-moment correlation test. In **a, c** and **d**, representative experimental results of three independent co-immunoprecipitation assessments are shown. In **b** and **h-k,** *n*=3 technical replicates, error bars, mean +/- SD, * P < 0.05, ** P < 0.01, *** P < 0.001, ns: non-significant, *P* values were assessed using two-tailed Student’s t tests. Source data are provided in Source Data file.

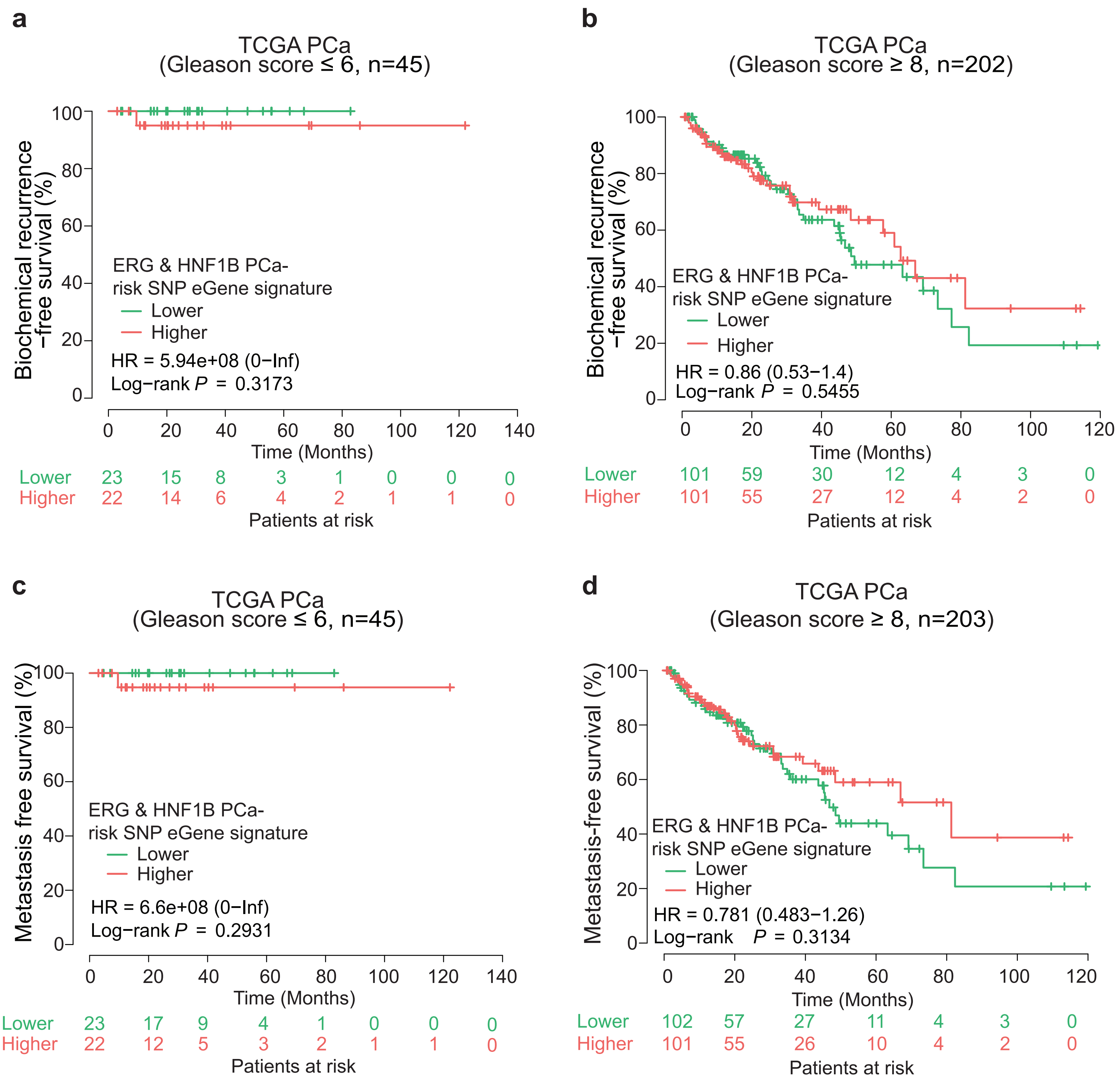
a
TCGA PCa
(Gleason score ≤ 6, n=45)
Biochemical recurrence −free survival (%)
ERG & HNF1B PCa-
risk SNP eGene signature
Lower
Higher
HR = 5.94e+08 (0−Inf)
Log−rank P = 0.3173
Time (Months)
Lower 23 15 8 3 1 0 0 0
Higher 22 14 6 4 2 1 1 0
Patients at risk
b
TCGA PCa
(Gleason score ≥ 8, n=202)
Biochemical recurrence −free survival (%)
ERG & HNF1B PCa-
risk SNP eGene signature
Lower
Higher
HR = 0.86 (0.53−1.4)
Log−rank P = 0.5455
Time (Months)
Lower 101 59 30 12 4 3 0
Higher 101 55 27 12 4 2 0
Patients at risk
c
TCGA PCa
(Gleason score ≤ 6, n=45)
Metastasis free survival (%)
ERG & HNF1B PCa-
risk SNP eGene signature
Lower
Higher
HR = 6.6e+08 (0−Inf)
Log−rank P = 0.2931
Time (Months)
Lower 23 17 9 4 1 0 0 0
Higher 22 12 5 3 2 1 1 0
Patients at risk
d
TCGA PCa
(Gleason score ≥ 8, n=203)
Metastasis-free survival (%)
ERG & HNF1B PCa-
risk SNP eGene signature
Lower
Higher
HR = 0.781 (0.483−1.26)
Log−rank P = 0.3134
Time (Months)
Lower 102 57 27 11 4 3 0
Higher 101 55 26 10 4 2 0
Patients at risk

Supplementary Fig. 9: No prognostic values were found for the ERG&HNF1B binding sites enriched PCa risk eQTL gene signature in patient groups with Gleason score 6 or 8.

a-d, Kaplan-Meier curves depicting biochemical relapse and metastatic rates of PCa patients in TCGA cohort with Gleason score ⩽ 6 (*n*=45) or ⩾ 8 (*n*=202). Patients were stratified by median z-score sum of HNF1B&ERG eQTL gene (eGene) signature score. *P* values were examined by a log-rank test. Source data are provided in Source Data file.

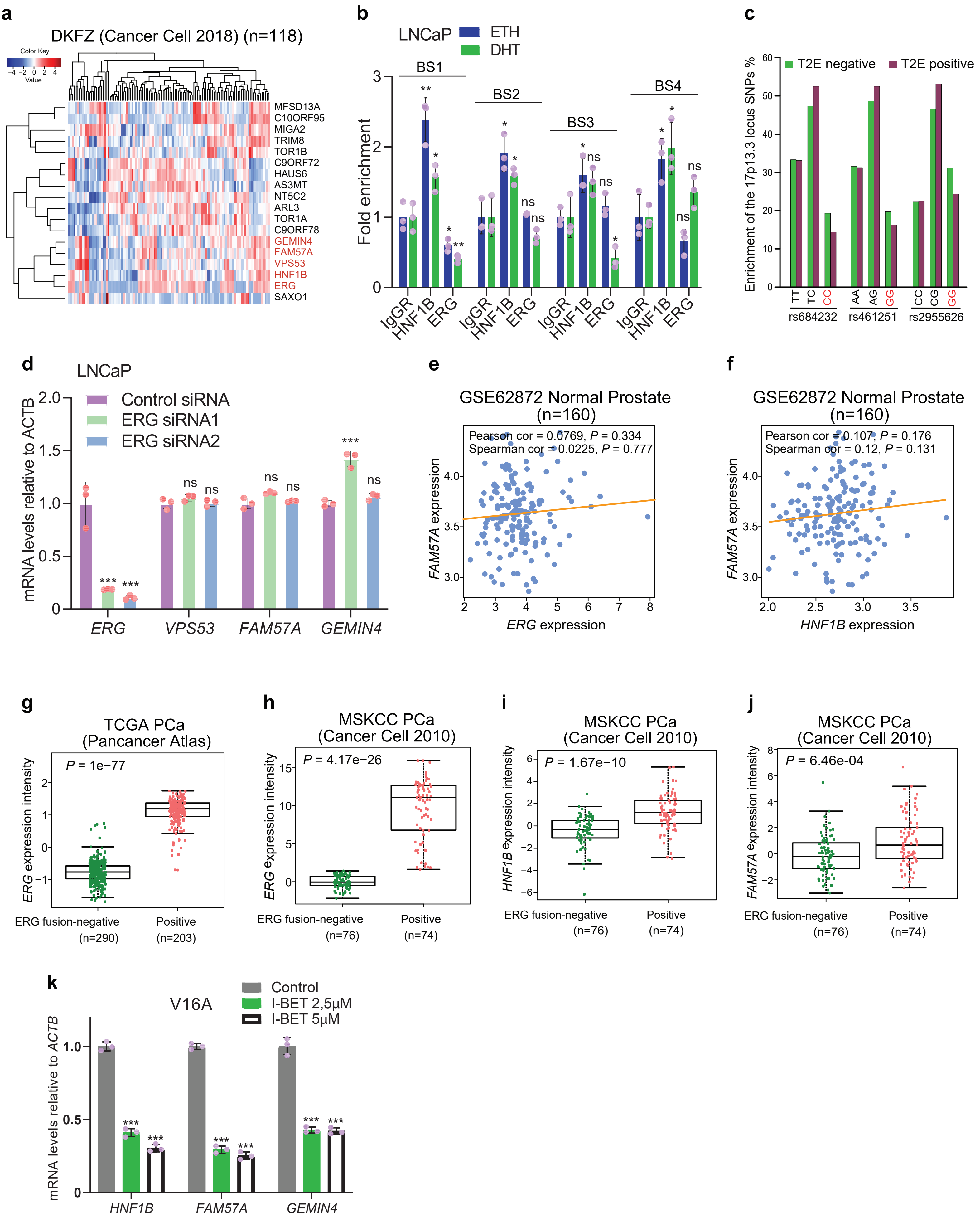
a
DKFZ (Cancer Cell 2018) (n=118)
Color Key
−4 −2 0 2 4
Value
MFSD13A
C10ORF95
MIGA2
TRIM8
TOR1B
C9ORF72
HAUS6
AS3MT
NT5C2
ARL3
TOR1A
C9ORF78
GEMIN4
FAM57A
VPS53
HNF1B
ERG
SAXO1
b
LNCaP
ETH
DHT
BS1
BS2
BS3
BS4
Fold enrichment
IgGR
HNF1B
ERG
c
T2E negative
T2E positive
Enrichment of the 17p13.3 locus SNPs %
TT TC CC
AA AG GG
CC CG GG
rs684232
rs461251
rs2955626
d
LNCaP
Control siRNA
ERG siRNA1
ERG siRNA2
mRNA levels relative to ACTB
ERG
VPS53
FAM57A
GEMIN4
e
GSE62872 Normal Prostate (n=160)
Pearson cor = 0.0769, P = 0.334
Spearman cor = 0.0225, P = 0.777
FAM57A expression
ERG expression
f
GSE62872 Normal Prostate (n=160)
Pearson cor = 0.107, P = 0.176
Spearman cor = 0.12, P = 0.131
FAM57A expression
HNF1B expression
g
TCGA PCa (Pancancer Atlas)
P = 1e−77
ERG expression intensity
ERG fusion-negative (n=290)
Positive (n=203)
h
MSKCC PCa (Cancer Cell 2010)
P = 4.17e−26
ERG expression intensity
ERG fusion-negative (n=76)
Positive (n=74)
i
MSKCC PCa (Cancer Cell 2010)
P = 1.67e−10
HNF1B expression intensity
ERG fusion-negative (n=76)
Positive (n=74)
j
MSKCC PCa (Cancer Cell 2010)
P = 6.46e-04
FAM57A expression intensity
ERG fusion-negative (n=76)
Positive (n=74)
k
V16A
Control
I-BET 2,5μM
I-BET 5μM
mRNA levels relative to ACTB
HNF1B
FAM57A
GEMIN4

Supplementary Fig. 10: Associations of the 17P13.3 locus risk genes with HNF1B and TMPRSS2-ERG fusion status in PCa clinical settings.

a, Heatmap displaying the expression levels of the HNF1B and ERG binding sites enriched PCa risk loci of the eQTL genes. Note that *FAM57A*, *GEMIN4* and *VPS53* show a similar expression pattern to HNF1B and ERG in the DKFZ cohort of PCa specimens (*n*=118). **b,** ChIP-qPCR validation of HNF1B and ERG binding at the regions within 17p13.3 in LNCaP cells treated with 100nM DHT and without (ETH-treated). *n*=6 samples; *P* values based on the order of appearance: 2,9E-03, 0,024, 0,034, 7,9E-03, 0,01, 0,027, 0,74, 0,18, 0,021, 0,064, 0,25, 0,036, 0,031, 0,013, 0,18, 0,082. **c,** Enrichment of the 17p13.3 locus SNPs in *TMPRSS2-ERG* fusion-positive and -negative tumours in the TCGA cohort. **d,** siRNA-ERG knockdown assays in LNCaP cells. *n*=3 samples; *P* values based on the order of appearance: 2,3E-03, 1,7E-03, 0,19, 0,84, 0,29, 0,52, 8,3E-04, 0,069. **e**, **f,** The expression levels of *FAM57A* and *ERG* or *HNF1B* are not significantly correlated in normal prostate glands (*n*=160). *P* values were assessed by the two-sided Pearson's product-moment correlation and Spearman's rank correlation rho tests. **g-j,** Expression levels of *ERG*, *HNF1B* and *FAM57A* are significantly elevated in the *TMPRSS2-ERG* fusion-positive PCa specimens compared to the *TMPRSS2-ERG* fusion-negative groups in the TCGA (*n*=493) and MSKCC (*n*=150) cohorts. *P* values were examined by the two-sided Mann–Whitney U test. The interquartile range (IQR) is depicted by the box with the median represented by the center line. Whiskers maximally extend to 1.5× IQR (with outliers shown). **k,** The mRNA levels of *HNF1B, FAM57A* and *GEMIN4* were downregulated in V16A cells treated with BRD4 antagonist I-BET for 24hrs. *n*=3 samples; *P* values based on the order of appearance: 1,6E-05, 7,4E-06, 2,7E-06, 2,5E-06, 8,5E-05, 8,4E-05. In **b**, **d** and **k**, *n*=3 technical replicates, error bars, mean +/- SD, * $P < 0.05$, ** $P < 0.01$, *** $P < 0.001$, ns: non-significant; two-tailed Student’s t tests. Source data are provided in Source Data file.

Supplementary Table 1.
Descriptive characteristics of Ruijin Biopsy cohort.

Characteristics	PCa Biopsy Group	Prostatectomy Group
Total number of patients	907	162
Age, yrs		
Median (IQR)	68 (63-75)	68.5 (64-73)
tPSA, ng/mL		
Median (IQR)	12.36 (7.58-25.05)	12.83 (7.48-27.92)
Prostate volume, mL		
Median (IQR)	81.55 (58.50-111.54)	63.95 (48.30-95.47)
ERG		
Negative	826 (91.1%)	123 (75.9%)
Positive	81 (8.9%)	39 (24.1%)
Family history of PCa		
Negative	884 (97.5%)	159 (98.1%)
Positive	23 (2.5%)	3 (1.9%)
Missing	0	0
Biopsy result		
Negative	489 (53.9%)	0
Positive	418 (46.1%)	162 (100%)
Grade Group		
1 (GS<=3+3)	95 (10.5%)	15 (9.3%)
2 (GS=3+4)	73 (8.0%)	55 (34.0%)
3 (GS=4+3)	80 (8.8%)	45 (27.8%)
4 (GS=8)	96 (10.6%)	10 (6.2%)
5 (GS=9 or 10)	60 (6.6%)	19 (11.7%)
Missing	8	18

Abbreviations: IQR, Interquartile range; tPSA, total prostate-specific antigen; PCa, prostate cancer; GS, Gleason Score.

Supplementary Table 2.
Association between PCa status and SNPs (791 PCa vs 752 non-PCa).

SNP	BP	Alt allele	Ref allele	Freq (alt)	Freq in gnomAD#	OR (95%CI)	P
rs11649743	36074979	A	G	0.32	0.34	0.78 (0.66-0.91)	1.89E-03
rs718960*	36077279	T	C	0.29	0.34	N/A	N/A
rs11263761	36097775	G	A	0.30	0.32	0.91 (0.77-1.07)	0.25
rs4430796	36098040	G	A	0.25	0.28	0.87 (0.74-1.03)	0.11
rs4239217	36098987	G	A	0.25	0.29	0.85 (0.72-1.01)	0.06
rs8064454	36101586	A	C	0.24	0.28	0.80 (0.68-0.95)	**0.01**
rs7405696	36102035	G	C	0.32	0.36	0.80 (0.68-0.95)	**8.89E-03**
rs11651052	36102381	A	G	0.24	0.28	0.82 (0.69-0.97)	**0.02**
rs9901746	36103149	G	A	0.32	0.38	0.81 (0.69-0.96)	**0.01**
rs11263763	36103565	G	A	0.24	0.26	0.80 (0.67-0.95)	**0.01**
rs11658063	36103872	C	G	0.23	0.26	0.81 (0.68-0.97)	**0.02**
rs12453443	36104121	C	G	0.32	0.38	0.82 (0.70-0.97)	**0.02**
rs3760511	36106313	T	G	0.31	0.36	0.86 (0.73-1.01)	0.06
**Additional**							
rs79882976	36083710	C	A	0.05	0.06	0.93 (0.65-1.33)	0.69
rs11651496	36084244	C	T	0.05	0.06	0.93 (0.65-1.33)	0.69
rs3744764	36090396	C	T	0.07	0.06	1.12 (0.84-1.50)	0.43

*This SNP failed to pass quality control (80%<genotype call <90%) in the new dataset.

#Frequency of alt allele in East Asian population by gnomAD dataset (https://gnomad.broadinstitute.org/).

Association results were performed by logistic regression model (additive effect) adjusted for patient's age at diagnosis. SNPs in HNF1B region could be grouped into 2-3 clusters based on the linkage disequilibrium. A two-tailed $p < 0.05/2$ or $0.05/3$ was considered statistically significant for multiplicity.

Abbreviation: N/A, not applicable; Freq, frequency; PCa, prostate cancer.

Supplementary Table 3.
Association between ERG expression and SNPs in PCa samples (136 ERG+ vs 655 ERG-).

SNP	BP	Alt allele	Ref allele	Freq (alt)	Freq in gnomAD#	OR (95%CI)	P
rs11649743	36074979	A	G	0.30	0.34	0.84 (0.63-1.12)	0.23
rs718960*	36077279	T	C	0.29	0.34	N/A	N/A
rs11263761	36097775	G	A	0.29	0.32	1.07 (0.80-1.43)	0.64
rs4430796	36098040	G	A	0.24	0.28	0.97 (0.72-1.31)	0.84
rs4239217	36098987	G	A	0.24	0.29	0.95 (0.71-1.29)	0.76
rs8064454	36101586	A	C	0.22	0.28	0.96 (0.70-1.30)	0.77
rs7405696	36102035	G	C	0.30	0.36	1.10 (0.82-1.47)	0.52
rs11651052	36102381	A	G	0.23	0.28	0.94 (0.69-1.28)	0.68
rs9901746	36103149	G	A	0.30	0.38	1.06 (0.78-1.42)	0.72
rs11263763	36103565	G	A	0.22	0.26	0.89 (0.64-1.23)	0.47
rs11658063	36103872	C	G	0.22	0.26	0.88 (0.64-1.22)	0.45
rs12453443	36104121	C	G	0.30	0.38	1.06 (0.78-1.42)	0.72
rs3760511	36106313	T	G	0.30	0.36	0.86 (0.73-1.01)	0.78
**Additional**							
rs79882976	36083710	C	A	0.05	0.06	2.00 (1.15-3.48)	**0.01**
rs11651496	36084244	C	T	0.05	0.06	2.00 (1.15-3.48)	**0.01**
rs3744764	36090396	C	T	0.07	0.06	1.67 (1.08-2.60)	**0.02**

*This SNP failed to pass quality control (80%<genotype call <90%) in the new dataset.

#Frequency of alt allele in East Asian population by gnomAD dataset (https://gnomad.broadinstitute.org/).

Association results were performed by logistic regression model (additive effect) adjusted for patient's age at diagnosis. SNPs in HNF1B region could be grouped into 2-3 clusters based on the linkage disequilibrium. A two-tailed $p < 0.05/2$ or $0.05/3$ was considered statistically significant for multiplicity.

Abbreviation: N/A, not applicable; Freq, frequency; PCa, prostate cancer.

Supplementary Table 4.
Experimental Models: Cell Lines.

Name	Source	Identifier
22Rv1	ATCC	Cat#CRL-2505
V16A	Hua et al. Cell 2018	
LNCaP	ATCC	Cat#CRL-1740
RWPE1	ATCC	Cat#CRL-11609
VCaP	ATCC	Cat#CRL-2876
293T	ATCC	Cat#CRL-11268
A549	ATCC	Cat#CCL-185
DU145	ATCC	Cat#HTB-81
PC3	ATCC	Cat#CRL-1435

Supplementary Table 5.
Gene cloning and related primers.

primer	Sequence 5' to 3'	target	restriction site	vector	experiment
rs718960.312BamHI-F	CGCGGATCCTCCTGGGCAATGTGGGGCTC	rs718960 region	BamHI/BamHI	pGL4.10[luc2]	Luciferase reporter assay
rs718960.312BamHI-R	CGCGGATCCGACCCAACAGGGCTGGGCTG				
rs7405696.403BamHI-F	CGCGGATCCAGCCCAACAATTGGGGAGGGTTT	rs7405696 region	BamHI/BamHI	pGL4.10[luc2]	Luciferase reporter assay
rs7405696.403BamHI-R	CGCGGATCCTTAACGAACGCGCTGCGGAC				
rs11651052.359BamHI-F	CGCGGATCCGTCCGCAGCGCGTTCGTTAAG	rs11651052 region	BamHI/BamHI	pGL4.10[luc2]	Luciferase reporter assay
rs11651052.359BamHI-R	CGCGGATCCCGGGAGCGCCGGACTTGATT				
rs9901746.383BamHI-F	CGCGGATCCGCCGCGTTTACAACTTCACCAA	rs9901746 region	BamHI/BamHI	pGL4.10[luc2]	Luciferase reporter assay
rs9901746.383BamHI-R	CGCGGATCCAGGCGGAAGGTGTGAGTCGC				
rs11263763.383.BamHI-F	CGCGGATCCGGGAGAAATCACCTCCGGGCG	rs11263763 region	BamHI/BamHI	pGL4.10[luc2]	Luciferase reporter assay
rs11263763.383.BamHI-R	CGCGGATCCCCAGGGCCGGGATCGTTTGT				
rs12453443.291BamHI-F	CGCGGATCCATAATTCCGGGCCTTGCATG	rs12453443 region	BamHI/BamHI	pGL4.10[luc2]	Luciferase reporter assay
rs12453443.291BamHI-R	CGCGGATCCAGCCCTTTGCCGGGAGGTTT				
HNF1Bpromoter(1)915.EcoRV-F	CGCTTGATATCGGAGCTTCACTTTGGCTACTCC	HNF1B promoter	EcoRV/HindIII	pGL4.10[luc2]	Luciferase reporter assay
HNF1Bpromoter(1)915.HindIII-R	CCCAAGCTTTTCCAAGGACGGAAAAAGAAG				
rs12453443G-F	CAGACCCGAGGCAGgGCGCAGGAAAAATG	rs12453443 region	NA	pGL4.10[luc2]	site-directed mutagenesis C→G
rs12453443G-R	CATTTTTCCTGCGCcCTGCCTCGGGTCTG				
rs9901746A-F	CGCCTGCGGCCGaGTGGGAGGGCCT	rs9901746 region	NA	pGL4.10[luc2]	site-directed mutagenesis G→A
rs9901746A-R	AGGCCCTCCCACtCGGCCGCAGGCG				
rs11263763A-F	CAGCCCCGAAATCGCaGCGCTTCAGTGTCCG	rs11263763 region	NA	pGL4.10[luc2]	site-directed mutagenesis G→A
rs11263763A-R	CGGACACTGAAGCGCtGCGATTTCGGGGCTG				
rs718960T-F	CAGGAATGTTCCTCtGGCTGGTGGACCTC	rs718960 region	NA	pGL4.10[luc2]	site-directed mutagenesis C→T
rs718960T-R	GAGGTCCACCAGCCaGAGGAACATTCCTG				
rs7405696C-F	AGGTTCCCTAACACcGAGAAAGGCACCGG	rs7405696 region	NA	pGL4.10[luc2]	site-directed mutagenesis G→C
rs7405696C-R	CCGGTGCCTTTCTCgGTGTTAGGGAACCT				
rs11651052G-F	GGTTTACTAGAACTCCgGGAACTCCAAAACGCG	rs11651052 region	NA	pGL4.10[luc2]	site-directed mutagenesis A→G
rs11651052G-R	CGCGTTTTGGAGTTCCcGGAGTTCTAGTAAACC				
GEMIN4pr(1)526EcoRV-F	CGCTTGATATCGGAACGCTGCTGTCTGGAGG	GEMIN4 promoter	EcoRV/HindIII	pGL4.10[luc2]	Luciferase reporter assay
GEMIN4pr(1)526HindIII-R	CCCAAGCTTGGCGGCGACGCCGGC				

VPS53pr(1)603EcoRV-F	CGCTTGATATCCTTCTGCGCAGAGCCCGG	VPS53 promoter	EcoRV/HindIII	pGL4.10[luc2]	Luciferase reporter assay
VPS53pr(1)603HindIII-R	CCCAAGCTTTCCGCCACCCGGCCC				
FAM57Apr(1)604EcoRV-F	CGCTTGATATCGGAGAGGCCACATCCTGAGG	FAM57A promoter	EcoRV/HindIII	pGL4.10[luc2]	Luciferase reporter assay
FAM57Apr(1)604HindIII-R	CCCAAGCTTCGGGGCTGCGGGTCCG				
rs684232 and rs461251.338BamHI-F	CGCGGATCCAAAGCCAGATGACGCAGC	rs684232 and rs461251 region	BamHI/BamHI	pGL4.10[luc2]	Luciferase reporter assay
rs684232 and rs461251.338BamHI-R	CGCGGATCCGGAGTTTCCCGGTTCACCT				
rs2955626.221BamHI-F	CGCGGATCCCAGCACAGCAACTCCCTC	rs2955626	BamHI/BamHI	pGL4.10[luc2]	Luciferase reporter assay
rs2955626.221BamHI-R	CGCGGATCCCCATCCTAAGGACGCGCTGA				
HNF1B-D-BamHI-F	CGGGATCCatggtgtccaagctcacgtcg	HNF1B sequence of D domain	BamHI/BstZ17I	pcDNA3.1/flag	Co-immunoprecipitation assay
HNF1B-D-BstZ17I-R	GCGTATACtgtgtcatagtcgtcgccgtcc				
HNF1B-POU-BamHI-F	CGGGATCCatgcctcccatcctcaaggagct	HNF1B sequence of POU domain	BamHI/BstZ17I	pcDNA3.1/flag	Co-immunoprecipitation assay
HNF1B-POU-BstZ17I-R	GCGTATACcagcttttgccggaatgcctc				
HNF1B-T-BamHI-F	CGGGATCCatggccatggacgcctatagctc	HNF1B sequence of T domain	BamHI/BstZ17I	pcDNA3.1/flag	Co-immunoprecipitation assay
HNF1B-T-BstZ17I-R	GCGTATACccaggcttgtagaggacactgt				
HNF1B-BamHI-F	CGGGATCCatggtgtccaagctcacgtcg	HNF1B	BamHI/BstZ17I	pcDNA3.1/flag	Co-immunoprecipitation assay
HNF1B-BstZ17I-R	GCGTATACccaggcttgtagaggacactgt				
ERG-KpnI-F	GGGGTACCatgattcagactgtcccggac	ERG	KpnI/XbaI	pcDNA3.1/V5	Co-immunoprecipitation assay
ERG-XbaI-R	GCTCTAGAgtagtaagtgcccagatgagaagg				
TMP-ERGKpnI-F	GGGGTACCtaggcgcgagctaagcaggag	TMPRSS2-ERG	KpnI/XbaI	pcDNA3.1/V5	Co-immunoprecipitation assay
TMP-ERG-XbaI-R	GCTCTAGAgtagtaagtgcccagatgagaagg				
HNF1BV5-F	AGCTTTGTTTAAACatggtgtccaagctcacgtc	HNF1B-V5	PmeI/BamHI	pLVET-IRES-GFP	Cell proliferation
HNF1BV5-R	CGGGATCCTCACGTAGAATCGAGACCG				

Supplementary Table 6.
Antibodies.

Name	Catalog #	Manufacturer	Application
Rabbit polyclonal anti- HNF1B	Cat#sc-22840X	Santa Cruz Biotechnology	ChIP/WB
Rabbit polyclonal IgG	Cat#sc-2027X	Santa Cruz Biotechnology	ChIP
Mouse monoclonal anti-V5	Cat#R960-25	Invitrogen (ThermoFisher)	co-IP/WB
Mouse monoclonal anti-V5-HRP	Cat#R961-25	Invitrogen (ThermoFisher)	WB
Mouse monoclonal anti-ERG	Cat#sc-376293	Santa Cruz Biotechnology	WB
Mouse monoclonal anti- HNF-1B	Cat#sc-130407	Santa Cruz Biotechnology	WB
Rabbit monoclonal IgG	Cat#ab172730	Abcam	ChIP/co-IP
Rabbit monoclonal anti-ERG	Cat#ab92513	Abcam	ChIP/co-IP/WB
Mouse monoclonal anti-FLAG	Cat#F1804	Sigma-aldrich	co-IP/WB
Goat anti-mouse IgG (H+L) secondary antibody, HRP	Cat#32430	Thermo Fisher	WB
Goat anti-rabbit IgG (H+L) secondary antibody, HRP	Cat#32460	Thermo Fisher	WB
Anti-rabbit Androgen Receptor	Cat#ab108341	Abcam	ChIP
H3K4me1	Cat#ab8895	Abcam	ChIP
H3K4me2	Cat#39141	ACTIVE MOTIF	ChIP
H3K4me3	Cat#ab12209	Abcam	ChIP
H3K27ac	Cat#ab4729	Abcam	ChIP
Mouse polyclonal IgG	Cat#sc-2025	Santa Cruz Biotechnology	ChIP

Supplementary Table 7.
Target sequences of siRNA and shRNA.

Name	Catalog #	Manufacturer	Sequence
Control-shRNA1	SHC002	Merck	CAACAAGATGAAGAGCACCAA
HNF1B-shRNA1	TRCN0000017508	Merck	CCGTACTGTCTATGTTGTGAT
HNF1B-shRNA2	TRCN0000017511	Merck	CCGACAATTCAACCAGACAGT
VPS53-shRNA1	TRCN0000180068	Merck	CCAGAAGTACCTCCGAGAATA
VPS53-shRNA2	TRCN0000148267	Merck	GATCAACCAAAGAAGCCTAAA
FAM57A-shRNA1	TRCN0000134609	Merck	GTGGTTTCTGATTCCATACAT
FAM57A-shRNA2	TRCN0000136978	Merck	CCTCAGACTTTGGGTATTGAT
GEMIN4-shRNA1	TRCN0000007892	Merck	GCAACCGTGTATCTGGACAAA
GEMIN4-shRNA2	TRCN0000007895	Merck	GTTTGTTTACACCCAGGTGTT
AllStars Hs Cell Death Control siRNA	SI04381048	Qiagen	
HNF1B-siRNA1	SI03040765	Qiagen	
HNF1B-siRNA2	SI03056956	Qiagen	
control siRNA	1027280	Qiagen	
ERG-siRNA1	SI03064726	Qiagen	
ERG-siRNA2	SI03089443	Qiagen	

Supplementary Table 8.
Oligonucleotides used in this study.

Experiment	primer description	Sequence 5' to 3'
RT-qPCR	hactin188RT-f	AGAAAATCTGGCACCACACC
RT-qPCR	hactin188RT-r	AGAGGCGTACAGGGATAGCA
RT-qPCR	GEMIN4-RT156f	ATGGACCTAGGACCCTTGAACAT
RT-qPCR	GEMIN4-RT156r	CTCCCTTAAGGCCTCCACGA
RT-qPCR	FAM57A-RT70f	AGCCTGCTCCAAGTACCCTT
RT-qPCR	FAM57A-RT70r	GAGGAGCTACGAGGAAGGCA
RT-qPCR	VPS53-RT69f	CCCTTGCTGCTGCTACAGTC
RT-qPCR	VPS53-RT69r	GAGCAAGAGTCGTCACGCAT
RT-qPCR	ERG exon56-F	CGCAGAGTTATCGTGCCAGCAGAT
RT-qPCR	ERG exon56-R	CCATATTCTTTCACCGCCCACTCC
RT-qPCR	HNF1BRT145-F	ACCAACAAGAAGATGCGCCG
RT-qPCR	HNF1BRT145-R	GCAAACATTCTGCCCTGTTGC
RT-qPCR	HNF1BRT92-F	AGGGCACCCCTATGAAGACC
RT-qPCR	HNF1BRT92-R	TGTCTGGTTGAATTGTCGGAGG
RT-qPCR	ATF5RT153-F	CCTGGCAGGTGATGGCTTCT
RT-qPCR	ATF5RT153-R	TCCTTCTTGAGGAGGGAGGC
RT-qPCR	GADD45ART92-F	CTCCTGCTCTTGGAGACCGA
RT-qPCR	GADD45ART92-R	TGTGGATTCGTCACCAGCAC
RT-qPCR	CENPART90-F	TCCTTAGGCGCTTCCTCCC
RT-qPCR	CENPART90-R	CAAGAGGTGTGTGCTCTTCTGA
RT-qPCR	EXO1RT244-F	GCTCGGCTAGGAATGTGCAG
RT-qPCR	EXO1RT244-R	TGGCCCGAATAAACCCGTTG
RT-qPCR	NR1D1RT224-F	CTGCCCAGCGTCATAACGAG
RT-qPCR	NR1D1RT224-R	GTTCATAGGACATGCCAGCAGAA
RT-qPCR	DIAPH3RT190-F	GCTTTTAAGTCTCAGTTTGGTGCC
RT-qPCR	DIAPH3RT190-R	GACCACTGAATGGCATCCGC
RT-qPCR	MCM10RT118-F	TGAAGAGTTGCAAGAGGAATTAAGG
RT-qPCR	MCM10RT118-R	AGGGGATTTTTGCAGACGGG
RT-qPCR	FBXO5RT242-F	AGATCGGGAGATGCTGAAGGAA
RT-qPCR	FBXO5RT242-R	TGTACAACTGGAATGCCCCC
RT-qPCR	SKA1RT146-F	ACGAAGGATACCAAAGGTCGT
RT-qPCR	SKA1RT146-R	GTAAGTCCTCCCCCTCGGAC
RT-qPCR	MAPRE1RT139-F	GCTCCTTCCCTTGTTGCTCC
RT-qPCR	MAPRE1RT139-R	GGTTCTTTCGCACCACACCA
RT-qPCR	SMARCC1RT226-F	GAAAGAAGCTCTAGAACAACAGAGG
RT-qPCR	SMARCC1RT226-R	GTAGGGAGGGGGCTGTTGAT
RT-qPCR	INCENPRT106-F	GAGCTGATGCCCAAAACACCT
RT-qPCR	INCENPRT106-R	TGCGGGATAACCTTCTCCTGAT
RT-qPCR	FLNBRT124-F	TGGTCCAGTCGGAGATTGGT
RT-qPCR	FLNBRT124-R	ATGCCACCATAACCTGCATCC
RT-qPCR	CDKN1ART171-F	AGGGGACAGCAGAGGAAGAC

RT-qPCR	CDKN1ART171-R	AGAAGATCAGCCGGCGTTTG
RT-qPCR	DCTN3RT151-F	GCTCACGGAAGGTGGCTGA
RT-qPCR	DCTN3RT151-R	GGTATGGCAATGCGGTCGAT
RT-qPCR	SHROOM1RT221-F	GCCCTTGGAGTTCCAGCATC
RT-qPCR	SHROOM1RT221-R	GGGGAACAGCCTGGACAATG
RT-qPCR	DUSP2RT187-F	CAGTCACTCGTCAGACCTGC
RT-qPCR	DUSP2RT187-R	CTTCACCCAGTCAATGAAGCCT
RT-qPCR	HIST1H2BCRT129-F	GCTCCAAGAAGGCAGTGACC
RT-qPCR	HIST1H2BCRT129-R	AAGAGATGCCAGTGTCGGGA
RT-qPCR	TGFB2RT119-F	CAACCCAGCGCTACATCGAC
RT-qPCR	TGFB2RT119-R	TCCCAGGTTCCTGTCTTTATGGT
ChIP-qPCR	TFF1RT85-F	TATGTAAAACAGTGGCTCCTGGC
ChIP-qPCR	TFF1RT85-R	CCAGACAGGTAAGGCGTGC
ChIP-qPCR	RASSF7RT88-F	GAGCCAGGTGAGGCGAGTA
ChIP-qPCR	RASSF7RT88-R	CACCAGCTTCCTCTTGCCAC
ChIP-qPCR	POLR3FRT84-F	CCGAAAGGCAAGGAAGGACC
ChIP-qPCR	POLR3FRT84-R	CGGGGAACCAGTGGAGGATA
ChIP-qPCR	CB1RT105-F	TTGCTAACGTTGAGGTGTGCAG
ChIP-qPCR	CB1RT105-R	CTGTGGACACGCTTTTTGCTTC
ChIP-qPCR	CB2RT88-F	GGCCCAGGTGTACTCACCATT
ChIP-qPCR	CB2RT88-R	CGGTTTTACAGCAAGTCTCCC
ChIP-qPCR	CB3RT89-F	TCACAAGGAGGCTTCTCCCC
ChIP-qPCR	CB3RT89-R	AAGCCTTTTGTTTCCTGCGG
ChIP-qPCR	BS1-140-F	GCAGCGACCTGGTGAGC
ChIP-qPCR	BS1-140-R	CACCTGACGTGACAGGAACG
ChIP-qPCR	BS2-127-F	CGCGTCGATGTGGAGAGGAT
ChIP-qPCR	BS2-127-R	GGGTGATCGGTGATCTGGCT
ChIP-qPCR	BS3-83-F	GCCCAGGGCGGAAAAGG
ChIP-qPCR	BS3-83-R	GAGGGGCTTCGGAAACTCG
ChIP-qPCR	BS4-149-F	GCTTAGGCCTGCTCACAACC
ChIP-qPCR	BS4-149-R	TAGTGAGCGACGTCCGGGTA
ChIP-qPCR	N.C-159-F	CTGGGCATGATGAGTGAGCG
ChIP-qPCR	N.C-159-R	TCGGGCCCTGTGACTGTTTA
ChIP-qPCR	rs12453443-215F	GAACTCGCCTCCCAGATCCC
ChIP-qPCR	rs12453443-215R	GACTGAAGGTGCGAGTGGGT
ChIP-qPCR	rs7405696-297F	AGCCGGGAGAGCTGGTTATT
ChIP-qPCR	rs7405696-297R	CGGGGGCGTCTAGAAAGTGG
ChIP-qPCR	rs718960-220F	CCACGATGTGGCTTTTGGCA
ChIP-qPCR	rs718960-220R	GAGCCCCAGCCAGTTGAGTA
ChIP-qPCR	rs11263763-272F	GGCTAGAGGGCAAACCGC
ChIP-qPCR	rs11263763-272R	CAGGGCCGGGATCGTTTGTG
ChIP-qPCR	rs11651052-276F	TCGAGGCCGCGAGAAAGG
ChIP-qPCR	rs11651052-276R	GGGAGCGCCGGACTTGATTA
ChIP-qPCR	CHIPneg-94F	GGCAGCAGAACATCACTGGG

ChIP-qPCR	CHIPneg-94R	ATGTCCTCCCCCACCAAGAG
3C-qPCR	ERCC3-Tagman probe	5'FAM-tagctctgatgtctgggaggc -3'TAM
3C-qPCR	ERCC3-3C reverse primer	aaatgcctcagccagtgaaag
3C-qPCR	ERCCC-3C constant primer	aggccttagtttgcctcagtt
3C-qPCR	ERCC3-F	acgtttattcctggtaactcaca
3C-qPCR	ERCC3-R	gagaattcagggatgcgcta
3C-qPCR	PROBE-R	5'FAM-GGCGCACGGGGAATTTCTAT-3'TAM
3C-qPCR	VPS,GEM,FAMCut site1-F	CCTAGCTGCTGGCTCTGTCT
3C-qPCR	VPS,GEM,FAMCut site1-R	CTCTGTGAGGTGGTTCCCTCT
3C-qPCR	VPS,GEM,FAMCut site2-F	AGAGGTAACTGCCCCGTCATA
3C-qPCR	VPS,GEM,FAMCut site2-R	AGCAGTTTTACAGAAGGCTCAG
3C-qPCR	VPS,GEM,FAMCut site3-F	CTGTCGAGACCTCAGGCT
3C-qPCR	VPS,GEM,FAMCut site3-R	GTTAGCCAGGTGTAGTAGTGTG
3C-qPCR	VPS,GEM,FAMCut site4-F	GACACTCACGAATTGGCCCTG
3C-qPCR	VPS,GEM,FAMCut site4-R	AAGCATATGAATGGCCCGTGAA
3C-qPCR	VPS,GEM,FAMCut site5-F	ACGTCCACCACCAAGAGAGA
3C-qPCR	VPS,GEM,FAMCut site5-R	CAAGTGTGAAGTGCCAGCCA
3C-qPCR	VPS,GEM,FAMCut site6-F	TTCTGCATGGCAGATGGTGG
3C-qPCR	VPS,GEM,FAMCut site6-R	TGTTCTTCTCGGTGGGCAAC
3C-qPCR	VPS,GEM,FAMCut site7-F	TCGGGAATGGAAAGGGCTTCTT
3C-qPCR	VPS,GEM,FAMCut site7-R	CACCGAGTTTCTCCTTTCCAACA
3C-qPCR	VPS,GEM,FAM-n.c.RT100-F	GAGCTTACCTCGAGATGGTGC
3C-qPCR	VPS,GEM,FAM-n.c.RT100-R	GTGCAAACACCAGCTACACC
3C-qPCR	VPS,GEM,FAMconstant-F1	CCCTCGGTCAGTGGCAG
3C-qPCR	VPSGEMFAM-R	GGCTGCAGTCACCTCGAAAA
CRISPR-Cas9	rs718960-gd1.1top	caccgGGCTCAGCTTGCCCACGATG
CRISPR-Cas9	rs718960-gd1.1bottom	aaacCATCGTGGGCAAGCTGAGCCc
CRISPR-Cas9	rs718960-gd1.2top	caccgAGTACTGGGTCCATCAAGCC
CRISPR-Cas9	rs718960-gd1.2bottom	aaacGGCTTGATGGACCCAGTACTc
CRISPR-Cas9	rs7405696-gd2.1top	caccgAACAAGACGCCTCGAACCTT
CRISPR-Cas9	rs7405696-gd2.1bottom	aaacAAGGTTCGAGGCGTCTTGTTc
CRISPR-Cas9	rs7405696-gd2.2top	caccgGCTCCTTAACGAACGCGCTG
CRISPR-Cas9	rs7405696-gd2.2bottom	aaacCAGCGCGTTCGTTAAGGAGCc
CRISPR-Cas9	rs11263763-gd3.1top	caccgGGGAGCCTGGTTCATCCGCC
CRISPR-Cas9	rs11263763-gd3.1bottom	aaacGGCGGATGAACCAGGCTCCCc
CRISPR-Cas9	rs11263763-gd3.2top	caccgAGGGTCCGGGTGGACACGCG
CRISPR-Cas9	rs11263763-gd3.2bottom	aaacCGCGTGTCCACCCGGACCCTc
CRISPR-Cas9	rs11651052-gd4.1top	caccgCAGGTGTCAAACGCCGGCCG
CRISPR-Cas9	rs11651052-gd4.1bottom	aaacCGGCCGGCGTTTGACACCTGc
CRISPR-Cas9	rs11651052-gd4.2top	caccgGCCGGAGACCCTCGCCGAGT
CRISPR-Cas9	rs11651052-gd4.2bottom	aaacACTCGGCGAGGGTCTCCGGCc
CRISPR-Cas9	HNF1B-gd1.1top	caccgTGCGCCGCAACCGGTTCAAA
CRISPR-Cas9	HNF1B-gd1.1bottom	aaacTTTGAACCGGTTGCGGCGCAc
CRISPR-Cas9	HNF1B-gd1.2top	caccgGTTGGAGCTATAGGCGTCCA

CRISPR-Cas9	HNF1B-gd1.2bottom	aaacTGGACGCCTATAGCTCCAACc
CRISPR-Cas9	rs4430796-gd1.1.1top	caccgAATTCTCATTGAATACAGAG
CRISPR-Cas9	rs4430796-gd1.1.1bottom	aaacCTCTGTATTCAATGAGAATTc
CRISPR-Cas9	rs4430796-gd1.1.2top	caccgATGCTGCATAAAGCTTAAAT
CRISPR-Cas9	rs4430796-gd1.1.2bottom	aaacATTTAAGCTTTATGCAGCATc
CRISPR-Cas9	rs4430796-gd1.2.1top	caccgGGAAACTTCTCAAAAGACAG
CRISPR-Cas9	rs4430796-gd1.2.1bottom	aaacCTGTCTTTTGAGAAGTTTCCc
CRISPR-Cas9	rs4430796-gd1.2.2top	caccgTCCAAAGACCCAACAACGCT
CRISPR-Cas9	rs4430796-gd1.2.2bottom	aaacAGCGTTGTTGGGTCTTTGGAc
CRISPR-Cas9	rs11263761-gd1.1.1top	caccgGCCTGTATAATAGTTACAGA
CRISPR-Cas9	rs11263761-gd1.1.1bottom	aaacTCTGTAACTATTATACAGGCc
CRISPR-Cas9	rs11263761-gd1.1.2top	caccgGAAAGTTTCTCAAGATGCTG
CRISPR-Cas9	rs11263761-gd1.1.2bottom	aaacCAGCATCTTGAGAAACTTTCc
CRISPR-Cas9	rs11263761-gd1.2.1top	caccgTCCTTCTGTAACTATTATAC
CRISPR-Cas9	rs11263761-gd1.2.1bottom	aaacGTATAATAGTTACAGAAGGAc
CRISPR-Cas9	rs11263761-gd1.2.2top	caccgATAACAACAATCTCTTGAAG
CRISPR-Cas9	rs11263761-gd1.2.2bottom	aaacCTTCAAGAGATTGTTGTTATc
CRISPR-Cas9	rs8064454-gd1.1.1top	caccgACGAGTTACAGTTTCAAACG
CRISPR-Cas9	rs8064454-gd1.1.1bottom	aaacCGTTTGAAACTGTAACTCGTc
CRISPR-Cas9	rs8064454-gd1.1.2top	caccgCAAGACGCCTCGAACCTTAG
CRISPR-Cas9	rs8064454-gd1.1.2bottom	aaacCTAAGGTTCGAGGCGTCTTGc
CRISPR-Cas9	rs8064454-gd1.2.1top	caccgAATGATCTGTGATGTTTACA
CRISPR-Cas9	rs8064454-gd1.2.1bottom	aaacTGTAAACATCACAGATCATTc
CRISPR-Cas9	rs8064454-gd1.2.2top	caccgTAGGCATACAAAATGAACAC
CRISPR-Cas9	rs8064454-gd1.2.2bottom	aaacGTGTTCATTTTGTATGCCTAc
CRISPR-Cas9	rs4239217-gd1.1.1top	caccgTCCCACTATCCTCCAAGAAC
CRISPR-Cas9	rs4239217-gd1.1.1bottom	aaacGTTCTTGGAGGATAGTGGGAc
CRISPR-Cas9	rs4239217-gd1.1.2top	caccgTGACTGATGGTCACGTATCA
CRISPR-Cas9	rs4239217-gd1.1.2bottom	aaacTGATACGTGACCATCAGTCAc
CRISPR-Cas9	rs4239217-gd1.2.1top	caccgCCCACTATCCTCCAAGAACA
CRISPR-Cas9	rs4239217-gd1.2.1bottom	aaacTGTTCTTGGAGGATAGTGGGc
CRISPR-Cas9	rs4239217-gd1.2.2top	caccgCTTCTTTGGAAATTGACTGA
CRISPR-Cas9	rs4239217-gd1.2.2bottom	aaacTCAGTCAATTTCCAAAGAAGc
CRISPR-Cas9	rs11658063-gd1.1.1top	caccgTCTCAGACCTCAGTTCGCAG
CRISPR-Cas9	rs11658063-gd1.1.1bottom	aaacCTGCGAACTGAGGTCTGAGAc
CRISPR-Cas9	rs11658063-gd1.1.2top	caccgTGTTCCATGCAGGGTCCGGG
CRISPR-Cas9	rs11658063-gd1.1.2bottom	aaacCCCGGACCCTGCATGGAACAc
CRISPR-Cas9	rs11658063-gd1.2.1top	caccgACCAGATCCTCTGCGAACTG
CRISPR-Cas9	rs11658063-gd1.2.1bottom	aaacCAGTTCGCAGAGGATCTGGTc
CRISPR-Cas9	rs11658063-gd1.2.2top	caccgAGGGTCCGGGTGGACACGCG
CRISPR-Cas9	rs11658063-gd1.2.2bottom	aaacCGCGTGTCCACCCGGACCCTc
CRISPR-Cas9	rs3760511-gd1.1.1top	caccgTCCCCTCCTGCCCGTCACCA
CRISPR-Cas9	rs3760511-gd1.1.1bottom	aaacTGGTGACGGGCAGGAGGGGAc
CRISPR-Cas9	rs3760511-gd1.1.2top	caccgGACCTGACCTGTAGAAGAAC

CRISPR-Cas9	rs3760511-gd1.1.2bottom	aaacGTTCTTCTACAGGTCAGGTCc
CRISPR-Cas9	rs3760511-gd1.2.1top	caccgGAAACTCCAATAGTGCTGCA
CRISPR-Cas9	rs3760511-gd1.2.1bottom	aaacTGCAGCACTATTGGAGTTTCc
CRISPR-Cas9	rs3760511-gd1.2.2top	caccgTCCCTGTTCTTCTACAGGTC
CRISPR-Cas9	rs3760511-gd1.2.2bottom	aaacGACCTGTAGAAGAACAGGGAc
CRISPR-Cas9	rs11649743-gd1.1.1top	caccgGTTCTGCCAGTCTCAGGTGT
CRISPR-Cas9	rs11649743-gd1.1.1bottom	aaacACACCTGAGACTGGCAGAACc
CRISPR-Cas9	rs11649743-gd1.1.2top	caccgTGAGAAGTGCATTAAGATTG
CRISPR-Cas9	rs11649743-gd1.1.2bottom	aaacCAATCTTAATGCACTTCTCAc
CRISPR-Cas9	rs11649743-gd1.2.1top	caccgAAAGCCCCAACACCTGAGAC
CRISPR-Cas9	rs11649743-gd1.2.1bottom	aaacGTCTCAGGTGTTGGGGCTTTc
CRISPR-Cas9	rs11649743-gd1.2.2top	caccgATCTTAATGCACTTCTCAAA
CRISPR-Cas9	rs11649743-gd1.2.2bottom	aaacTTTGAGAAGTGCATTAAGATc
CRISPR-Cas9	rs9901746-gd1.1top	caccgAACTGCAGCACGTGGAGTTG
CRISPR-Cas9	rs9901746-gd1.1bottom	aaacCAACTCCACGTGCTGCAGTTc
CRISPR-Cas9	rs9901746-gd1.2top	caccgTTTATGTTACGTCTCAGCTG
CRISPR-Cas9	rs9901746-gd1.2bottom	aaacCAGCTGAGACGTAACATAAAc
CRISPR-Cas9	rs9901746-gd2.1top	caccgTAGGAGCGGCTGGGGCGCCA
CRISPR-Cas9	rs9901746-gd2.1bottom	aaacTGGCGCCCCAGCCGCTCCTAc
CRISPR-Cas9	rs9901746-gd2.2top	caccgCAGGTCCTGAGCGACCTCCC
CRISPR-Cas9	rs9901746-gd2.2bottom	aaacGGGAGGTCGCTCAGGACCTGc
CRISPR-Cas9	rs12453443-gd1.1top	caccgGTTCTTTGCCGAAGGACAGG
CRISPR-Cas9	rs12453443-gd1.1bottom	aaacCCTGTCCTTCGGCAAAGAACc
CRISPR-Cas9	rs12453443-gd1.2top	caccgGATCTGGGAGGCGAGTTCCC
CRISPR-Cas9	rs12453443-gd1.2bottom	aaacGGGAACTCGCCTCCCAGATCc
CRISPR-Cas9	rs12453443-gd2.1top	caccgGTGGGCAAACCTCCCGGCAA
CRISPR-Cas9	rs12453443-gd2.1bottom	aaacTTGCCGGGAGGTTTGCCCACc
CRISPR-Cas9	rs12453443-gd2.2top	caccgCAGGAAAAATGCGCGGCCTG
CRISPR-Cas9	rs12453443-gd2.2bottom	aaacCAGGCCGCGCATTTTTCCTGc
CRISPR-Cas9	rs461251-gd1.1.1top	caccgTTATCAATGGTGGACTATTC
CRISPR-Cas9	rs461251-gd1.1.1bottom	aaacGAATAGTCCACCATTGATAAc
CRISPR-Cas9	rs461251-gd1.1.2top	caccgATCCTCTCCACATCGACGCG
CRISPR-Cas9	rs461251-gd1.1.2bottom	aaacCGCGTCGATGTGGAGAGGATc
CRISPR-Cas9	rs461251-gd1.2.1top	caccgCAGGTGAACCGGGAAACTCC
CRISPR-Cas9	rs461251-gd1.2.1bottom	aaacGGAGTTTCCCGGTTCACCTGc
CRISPR-Cas9	rs461251-gd1.2.2top	caccgTGGTGGAGCGGGTCCTCCTT
CRISPR-Cas9	rs461251-gd1.2.2bottom	aaacAAGGAGGACCCGCTCCACCAc
CRISPR-Cas9	rs2955626-gd2.1.1top	caccgACTCCCTCGCGGCAGCGACC
CRISPR-Cas9	rs2955626-gd2.1.1bottom	aaacGGTCGCTGCCGCGAGGGAGTc
CRISPR-Cas9	rs2955626-gd2.1.2top	caccgACAGAGCGGCTGACGGAGCC
CRISPR-Cas9	rs2955626-gd2.1.2bottom	aaacGGCTCCGTCAGCCGCTCTGTc
CRISPR-Cas9	rs2955626-gd2.2.1top	caccgGCGCGTCCTTAGGATGGGCG
CRISPR-Cas9	rs2955626-gd2.2.1bottom	aaacCGCCCATCCTAAGGACGCGCc
CRISPR-Cas9	rs2955626-gd2.2.2top	caccgCGCCGGGATACTGCGTTGCC

CRISPR-Cas9	rs2955626-gd2.2.2bottom	aaacGGCAACGCAGTATCCCGGCGc
CRISPR-Cas9	rs684232-gd3.1.1top	caccgAGCTGGGGAGACGTTTACTG
CRISPR-Cas9	rs684232-gd3.1.1bottom	aaacCAGTAAACGTCTCCCCAGCTc
CRISPR-Cas9	rs684232-gd3.1.2top	caccgCTCCAGGGCGCTGCGTCATC
CRISPR-Cas9	rs684232-gd3.1.2bottom	aaacGATGACGCAGCGCCCTGGAGc
CRISPR-Cas9	rs684232-gd1.1.1top	caccgTTATCAATGGTGGACTATTC
CRISPR-Cas9	rs684232-gd1.1.1bottom	aaacGAATAGTCCACCATTGATAAc
CRISPR-Cas9	rs684232-gd1.1.2top	caccgATCCTCTCCACATCGACGCG
CRISPR-Cas9	rs684232-gd1.1.2bottom	aaacCGCGTCGATGTGGAGAGGATc

Supplementary Table 9.
Recombinant DNA.

Name	Source	Identifier
pGEN-MCS-Renilla	Wei et al., 2010	N/A
pcDNA3.1/V5-His A	Invitrogen	Cat#V81020
pcDNA3.1/flag	designed for this study	N/A
pLVET-IRES-GFP	Zhang et al., 2017	N/A
pLKO.1 Puro	Stewart et al., 2003	Addgene #8453
pSpCas9 (BB)-2A-Puro (PX459)	Feng Zhang Lab at MIT	N/A
pGL4.10[luc2]	Promega	Cat#E665A
pVSVG-envelope	addgene	Cat#14888
pMDLg/pRRE-packaging	addgene	Cat#12251
pRSV-Rev-packaging	addgene	Cat#12253
pGL4.75[hRluc/CMV]	Promega	Cat#E693A

Supplementary Table 10.
Software and Algorithms.

Name	Source	Identifier
HaploReg v4.1	Ward and Kellis, 2012	http://archive.broadinstitute.org/mammals/haploreg/haploreg.php
FastQC	Babraham Bioinformatics Institute	https://www.bioinformatics.babraham.ac.uk/projects/fastqc/
Matrix eQTL	R Bioconductor	https://bioconductor.org/
Trimmomatic	Bolger et al., 2014	http://www.usadellab.org/cms/?page=trimmomatic
Tophat2	Center for Computational Biology at Johns Hopkins University	https://ccb.jhu.edu/software/tophat/index.shtml
HTSeq	Bioconductor	https://htseq.readthedocs.io/en/release_0.9.1/
DESeq2	Love et al., 2014	https://bioconductor.org/packages/release/bioc/html/DESeq2.html
GSEA	Subramanian et al., 2005	http://software.broadinstitute.org/gsea/index.jsp
MACS2	Zhang et al., 2008	https://github.com/macs3-project/MACS
HOMER V4.10	Heinz et al., 2010	http://homer.ucsd.edu/homer/
IGV 2.4.10	Robinson et al., 2011	https://software.broadinstitute.org/software/igv/
ChIPseeker 1.18.0	Yu et al., 2015	https://www.bioconductor.org/packages/release/bioc/html/ChIPseeker.html
R Version 3.6.3	R	https://www.r-project.org/
CRISPR design tool	Feng Zhang Lab at MIT	http://crispr.mit.edu/

Supplementary Table 11.
Deposited Data.

Name	Source	Identifier
Raw RNA-seq data	This paper	
Oncomine database	Rhodes et al., 2004	https://www.oncomine.org/resource/login.html
cDNA microarray assay of gene expression profiling	Welsh et al., 2001	https://www.oncomine.org/resource/login.html
cDNA microarray assay of gene expression profiling	Vanaja et al., 2003	https://www.oncomine.org/resource/login.html
cDNA microarray assay of gene expression profiling	Glinsky et al., 2004	https://www.oncomine.org/resource/login.html
cDNA microarray assay of gene expression profiling	Luo et al., 2001	https://www.oncomine.org/resource/login.html
cDNA microarray assay of gene expression profiling	Yu et al., 2004	GEO: GSE6919
cDNA microarray assay of gene expression profiling	Taylor et al., 2010	GEO: GSE21032
cDNA microarray assay of gene expression profiling	Bittner et al., 2005	GEO: GSE2109
cDNA microarray assay of gene expression profiling	Wallace et al., 2008	GEO: GSE6956
cDNA microarray assay of gene expression profiling	Penney et al., 2014	GEO: GSE62872
cDNA microarray assay of gene expression profiling	Ross-Adams et al., 2015	GEO: GSE70769
cDNA microarray assay of gene expression profiling	Ross-Adams et al., 2015	GEO: GSE70768
cDNA microarray assay of gene expression profiling	Lapointe et al., 2004	GEO: GSE3933
Processed RNA-seq data	Cancer Genome Atlas Research Network, 2015	http://www.cbioportal.org/
Processed ChIP-seq data	Mei et al., 2017	http://cistrome.org/db/#/
GWAS catelog	Buniello et al., 2019	https://www.ebi.ac.uk/gwas/
Genome-wide CRISPR/Cas9 screening of cancer cell survival genes	DepMap, Broad, 2019	https://depmap.org/portal/achilles/

Supplementary Table 12.
Critical Commercial Assays.

Name	Source	Identifier
RNeasy Mini Kit	QIAGEN	Cat#74106
RNase-Free DNase	QIAGEN	Cat#79254
High-Capacity cDNA Reverse Transcription Kit	Applied Biosystems	Cat#4368814
GeneJET PCR Purification Kit	Thermo Scientific	Cat#K0701
GeneJET Gel Extraction Kit	Thermo Scientific	Cat#K0691
PureYield Plasmid Miniprep System	Promega	Cat#A1223
Dual-Glo Luciferase Assay System	Promega	Cat#E2940
MinElute PCR Purification Kit	QIAGEN	Cat#28006
iScript Reverse Transcription Supermix	Bio-rad	Cat#1708840

Supplementary Table 13.
SNP genomic locations in the 17q12 and 17p13.3 loci.

SNPs	Chr	Position	hg19 SNP-centered region	Gene
rs4430796	17	36098040	chr17:36097540-36098540	HNF1B
rs11263761	17	36097775	chr17:36097275-36098275	HNF1B
rs11263763	17	36103565	chr17:36103065-36104065	HNF1B
rs11651052	17	36102381	chr17:36101881-36102881	HNF1B
rs8064454	17	36101586	chr17:36101086-36102086	HNF1B
rs9901746	17	36103149	chr17:36102649-36103649	HNF1B
rs12453443	17	36104121	chr17:36103621-36104621	HNF1B
rs7405696	17	36102035	chr17:36101535-36102535	HNF1B
rs4239217	17	36098987	chr17:36098487-36099487	HNF1B
rs11658063	17	36103872	chr17:36103372-36104372	HNF1B
rs3760511	17	36106313	chr17:36105813-36106813	HNF1B
rs11649743	17	36074979	chr17:36074479-36075479	HNF1B
rs718960	17	36077279	chr17:36076779-36077779	HNF1B
rs2955626	17	618100	chr17:617600-618600	VPS53, FAM57A, GEMIN4
rs684232	17	618965	chr17:618465-619465	VPS53, FAM57A, GEMIN4
rs461251	17	619162	chr17:618162-619662	VPS53, FAM57A, GEMIN4

Supplementary Table 14.
Bacterial and Virus Strains.

Name	Source	Identifier
DH5α Chemically Competent cells	This paper	N/A
Stbl3 Chemically Competent cells	This paper	N/A
Lentivirus	This paper	N/A

Supplementary Table 15.
Chemicals, Peptides, and Recombinant Proteins.

Name	Source	Identifier
5α-Dihydrotestosterone (DHT) solution	From Olli A. Jänne lab (University of Helsinki)	N/A
BamHI-HF	New England Biolabs	Cat#R3136M
BstZ17I-HF	New England Biolabs	Cat#R3594S
KpnI-HF	New England Biolabs	Cat#R3142M
PmeI	New England Biolabs	Cat#R0560S
XbaI	New England Biolabs	Cat#R0145M
HindIII-HF	New England Biolabs	Cat#R3104M
EcoRV-HF	New England Biolabs	Cat#R3195M
BbsI	New England Biolabs	Cat#R0539S
CutSmart Buffer	New England Biolabs	Cat#B7204S
T4 DNA Ligase	New England Biolabs	Cat#M0202M
T4 DNA Ligase Reaction Buffer	New England Biolabs	Cat#B0202S
SYBR Master Mix	Applied Biosystems	Cat#4472920
Fetal Bovine Serum	Thermo Fisher	Cat#16000044
Penicillin-Streptomycin	Thermo Fisher	Cat#15140122
Keratinocyte-Serum Free Medium	Invitrogen	Cat#17005-042
Lipofectamine 3000	Thermo Fisher	Cat#L3000015
Lipofectamine 2000	Thermo Fisher	Cat#11668019
cOmplete, Mini, EDTA-free Protease Inhibitor Cocktail	Roche	Cat#04693159001
Dynabead protein G	Invitrogen	Cat#10004D
X-treme GENE HP DNA Transfection Reagent	Roche	Cat#06366236001
HiPerFect Transfection Reagent	QIAGEN	Cat#301705
high glucose, DMEM	Thermo Fisher	Cat#11965092
Low glucose DMEM	Thermo Fisher	Cat#11885084
RPMI-1640 Medium	Sigma-Aldrich	Cat#R8758
F-12K	ATCC	Cat#30-2004
Trypsin-EDTA (0.25%), phenol red	Thermo Fisher	Cat#25200-072
Cell Proliferation Kit II	Roche	Cat#11465015001
Polybrene	Merck	Cat#H9268
Puromycin	Merck	Cat#P9620
2x Phusion Master Mix with HF Buffer	Thermo Fisher	Cat#F531
Quanti tech probe PCR mix	QIAGEN	Cat#204343
Pierce BCA Protein Assay Kit	Thermo Fisher	Cat#23225
Pierce lane marker reducing sample buffer	Thermo Scientific	Cat#39000
Opti-MEM I Reduced-Serum medium	Thermo Fisher	Cat#11058021
Lumi-Light Western Blotting Substrate	Merck	Cat#12015200001
SuperSignal West Femto Maximum Sensitivity Substrate	Thermo Fisher	Cat#34094
I-BET-CAS 1260907-17-2 -Calbiochem	Sigma-Aldrich	Cat#401010-5MG
EMEM	ATCC	Cat#30-2003
Unstained Protein Ladder (10 - 200 kDa)	abcam	Cat#ab234618
PageRuler™ Prestained Protein Ladder, 10 to 180 kDa	Thermo Scientific	Cat# 26616
Precision Plus Protein Dual Color Standards	BIO-RAD	Cat# 1610374
