# Supplementary material for: Extensive germline-somatic interplay contributes to prostate cancer progression through HNF1B co-option of TMPRSS2-ERG
Source: Nat Commun. 2022 Nov 28;13:7320. doi: 10.1038/s41467-022-34994-z (PMC9705428; doi:10.1038/s41467-022-34994-z)
Supplement: Supplementary file 3 — Description of Additional Supplementary Files [file 41467_2022_34994_MOESM3_ESM.pdf]

File Name: Supplementary Data 1

Description: cis-eQTL analysis in this Wisconsin cohort exhibited the strongest association of HNF1B among all other genes within the one mega-base window of the 11 SNPs.
