# Supplementary material for: Extensive germline-somatic interplay contributes to prostate cancer progression through HNF1B co-option of TMPRSS2-ERG
Source: Nat Commun. 2022 Nov 28;13:7320. doi: 10.1038/s41467-022-34994-z (PMC9705428; doi:10.1038/s41467-022-34994-z)
Supplement: Supplementary file 5 — Reporting Summary [file 41467_2022_34994_MOESM5_ESM.pdf]

nature portfolio

Corresponding author(s): Gong-Hong Wei

Last updated by author(s): Oct 25, 2022

Reporting Summary

Nature Portfolio wishes to improve the reproducibility of the work that we publish. This form provides structure for consistency and transparency in reporting. For further information on Nature Portfolio policies, see our Editorial Policies and the Editorial Policy Checklist.

Statistics

For all statistical analyses, confirm that the following items are present in the figure legend, table legend, main text, or Methods section.

n/a	Confirmed	
☐	☒	The exact sample size (*n*) for each experimental group/condition, given as a discrete number and unit of measurement
☐	☒	A statement on whether measurements were taken from distinct samples or whether the same sample was measured repeatedly
☐	☒	The statistical test(s) used AND whether they are one- or two-sided<br>*Only common tests should be described solely by name; describe more complex techniques in the Methods section.*
☐	☒	A description of all covariates tested
☐	☒	A description of any assumptions or corrections, such as tests of normality and adjustment for multiple comparisons
☐	☒	A full description of the statistical parameters including central tendency (e.g. means) or other basic estimates (e.g. regression coefficient) AND variation (e.g. standard deviation) or associated estimates of uncertainty (e.g. confidence intervals)
☐	☒	For null hypothesis testing, the test statistic (e.g. *F*, *t*, *r*) with confidence intervals, effect sizes, degrees of freedom and *P* value noted<br>*Give P values as exact values whenever suitable.*
☒	☐	For Bayesian analysis, information on the choice of priors and Markov chain Monte Carlo settings
☐	☒	For hierarchical and complex designs, identification of the appropriate level for tests and full reporting of outcomes
☐	☒	Estimates of effect sizes (e.g. Cohen's *d*, Pearson's *r*), indicating how they were calculated

Our web collection on statistics for biologists contains articles on many of the points above.

Software and code

Policy information about availability of computer code

Data collection	No software was used for data collection in this study
Data analysis	bedtools v.2.27.1<br>Bowtie2 v.2.4.4<br>ChIPseeker v.1.18.0<br>clusterProfiler v.3.14.3<br>deepTools v.3.3.2<br>DESeq2 v.1.16.1<br>enrichplot v.1.12.0<br>FastQC v.0.11.4<br>Gene Set Enrichment Analysis (GSEA) v.4.0.3<br>Haploreg v.4.1<br>Haploreg v.4.1.<br>HOMER v.4.11<br>HTSeq v.0.11.0 (htseq-count)<br>IGV v.2.4.10<br>MACS2 v. 2.2.7.1<br>MatrixEQTL v.2.2<br>metafor v.3.4.0<br>pheatmap v1.0.12<br>R environment v.4.2.0<br>R version v. 3.6.3<br>RStudio v.1.2.5033

samtools v.1.9
SortMeRna
Survival (v. 3.2.3)
TopHat2 v.2.1.1
Trimmomatic v.0.39
UCSC
CRISPOR v.4.99
BLASTn

For manuscripts utilizing custom algorithms or software that are central to the research but not yet described in published literature, software must be made available to editors and reviewers. We strongly encourage code deposition in a community repository (e.g. GitHub). See the Nature Portfolio guidelines for submitting code & software for further information.

Data

Policy information about availability of data

All manuscripts must include a data availability statement. This statement should provide the following information, where applicable:

- Accession codes, unique identifiers, or web links for publicly available datasets
- A description of any restrictions on data availability
- For clinical datasets or third party data, please ensure that the statement adheres to our policy

All data used in this manuscript were publicly available. RNA-seq or microarray data were retrieved from public databases including cBioPortal for Cancer Genomics, Oncomine database and GEO database. The ERG ChIP-seq profiling data in VCaP cells were obtained from the Cistrome Data Browser (GEO: GSM717395, GSM717396, GSM717397, GSM1193658, GSM1328978, GSM1328980, GSM1328981, GSM2086315, GSM2086314, GSM2086313, GSM2086312, GSM2086311, GSM2086310, GSM2086309, GSM353637). The data used for the eQTL analyses described in this manuscript were obtained from GTEx portal, PancanQTL and ncRNA-eQTL. The FinnGen data used in this research are publicly available to qualified researchers and detailed documentation is provided on the FinnGen study website (https://www.finngen.fi/). The HNF1B RNA-seq and ChIP-seq data generated in this study have been deposited in the ENA (European Nucleotide Archive) database (https://www.ebi.ac.uk/ena/browser/home) under accession codes PRJEB46082 and PRJEB46088, respectively. The ChIP-seq data for HNF1B and ERG replicates generated in this study are available at ENA under accession code PRJEB49662. Moreover, we have also clearly indicated the source of those publicly available data used in the manuscript. The authors declare that all the other data supporting the findings of this study are available within the Article, Supplementary Information or Source Data file. There is no restrictions on data availability.

Field-specific reporting

Please select the one below that is the best fit for your research. If you are not sure, read the appropriate sections before making your selection.

☒ Life sciences ☐ Behavioural & social sciences ☐ Ecological, evolutionary & environmental sciences

For a reference copy of the document with all sections, see nature.com/documents/nr-reporting-summary-flat.pdf

Life sciences study design

All studies must disclose on these points even when the disclosure is negative.

Sample size	No sample size calculation was performed. Sample size was determined from similar experiments in the literature and for all experiments a minimum of three technical replicates were analyzed per sample.
Data exclusions	No data were excluded.
Replication	All attempts in replication were successful. All the experiments were performed in biological replicates to ensure reproducibility of the data.We have performed 2 biological replicates of siControl, siRNA1 HNF1B and siRNA2 HNF1B for RNA-seq. One biological replicate per ChIP-seq sample.
Randomization	For the cell studies, the treated groups were attributed randomly.
Blinding	ChIP-seq and ChIP-qPCR wet lab experiments were performed by the two first co-authors. In general, blinding was not required as each experiment was designed to give an explicit outcome which is independent on the judgement of the researchers.

Behavioural & social sciences study design

All studies must disclose on these points even when the disclosure is negative.

Study description	*Briefly describe the study type including whether data are quantitative, qualitative, or mixed-methods (e.g. qualitative cross-sectional, quantitative experimental, mixed-methods case study).*
Research sample	*State the research sample (e.g. Harvard university undergraduates, villagers in rural India) and provide relevant demographic information (e.g. age, sex) and indicate whether the sample is representative. Provide a rationale for the study sample chosen. For studies involving existing datasets, please describe the dataset and source.*

Sampling strategy	*Describe the sampling procedure (e.g. random, snowball, stratified, convenience). Describe the statistical methods that were used to predetermine sample size OR if no sample-size calculation was performed, describe how sample sizes were chosen and provide a rationale for why these sample sizes are sufficient. For qualitative data, please indicate whether data saturation was considered, and what criteria were used to decide that no further sampling was needed.*
Data collection	*Provide details about the data collection procedure, including the instruments or devices used to record the data (e.g. pen and paper, computer, eye tracker, video or audio equipment) whether anyone was present besides the participant(s) and the researcher, and whether the researcher was blind to experimental condition and/or the study hypothesis during data collection.*
Timing	*Indicate the start and stop dates of data collection. If there is a gap between collection periods, state the dates for each sample cohort.*
Data exclusions	*If no data were excluded from the analyses, state so OR if data were excluded, provide the exact number of exclusions and the rationale behind them, indicating whether exclusion criteria were pre-established.*
Non-participation	*State how many participants dropped out/declined participation and the reason(s) given OR provide response rate OR state that no participants dropped out/declined participation.*
Randomization	*If participants were not allocated into experimental groups, state so OR describe how participants were allocated to groups, and if allocation was not random, describe how covariates were controlled.*

Ecological, evolutionary & environmental sciences study design

All studies must disclose on these points even when the disclosure is negative.

Study description	*Briefly describe the study. For quantitative data include treatment factors and interactions, design structure (e.g. factorial, nested, hierarchical), nature and number of experimental units and replicates.*
Research sample	*Describe the research sample (e.g. a group of tagged Passer domesticus, all Stenocereus thurberi within Organ Pipe Cactus National Monument), and provide a rationale for the sample choice. When relevant, describe the organism taxa, source, sex, age range and any manipulations. State what population the sample is meant to represent when applicable. For studies involving existing datasets, describe the data and its source.*
Sampling strategy	*Note the sampling procedure. Describe the statistical methods that were used to predetermine sample size OR if no sample-size calculation was performed, describe how sample sizes were chosen and provide a rationale for why these sample sizes are sufficient.*
Data collection	*Describe the data collection procedure, including who recorded the data and how.*
Timing and spatial scale	*Indicate the start and stop dates of data collection, noting the frequency and periodicity of sampling and providing a rationale for these choices. If there is a gap between collection periods, state the dates for each sample cohort. Specify the spatial scale from which the data are taken*
Data exclusions	*If no data were excluded from the analyses, state so OR if data were excluded, describe the exclusions and the rationale behind them, indicating whether exclusion criteria were pre-established.*
Reproducibility	*Describe the measures taken to verify the reproducibility of experimental findings. For each experiment, note whether any attempts to repeat the experiment failed OR state that all attempts to repeat the experiment were successful.*
Randomization	*Describe how samples/organisms/participants were allocated into groups. If allocation was not random, describe how covariates were controlled. If this is not relevant to your study, explain why.*
Blinding	*Describe the extent of blinding used during data acquisition and analysis. If blinding was not possible, describe why OR explain why blinding was not relevant to your study.*

Did the study involve field work? ☐ Yes ☐ No

Field work, collection and transport

Field conditions	*Describe the study conditions for field work, providing relevant parameters (e.g. temperature, rainfall).*
Location	*State the location of the sampling or experiment, providing relevant parameters (e.g. latitude and longitude, elevation, water depth).*
Access & import/export	*Describe the efforts you have made to access habitats and to collect and import/export your samples in a responsible manner and in compliance with local, national and international laws, noting any permits that were obtained (give the name of the issuing authority, the date of issue, and any identifying information).*
Disturbance	*Describe any disturbance caused by the study and how it was minimized.*

Reporting for specific materials, systems and methods

We require information from authors about some types of materials, experimental systems and methods used in many studies. Here, indicate whether each material, system or method listed is relevant to your study. If you are not sure if a list item applies to your research, read the appropriate section before selecting a response.

Materials & experimental systems

n/a	Involved in the study
☐	☒ Antibodies
☐	☒ Eukaryotic cell lines
☒	☐ Palaeontology and archaeology
☒	☐ Animals and other organisms
☐	☒ Human research participants
☒	☐ Clinical data
☒	☐ Dual use research of concern

Methods

n/a	Involved in the study
☐	☒ ChIP-seq
☒	☐ Flow cytometry
☒	☐ MRI-based neuroimaging

Antibodies

Antibodies used

Rabbit polyclonal anti- HNF1B, Cat#sc-22840X , Santa Cruz Biotechnology, ChIP/WB , Lot F3016, RRID:AB_2279595

Rabbit polyclonal IgG, Cat#sc-2027X, Santa Cruz Biotechnology, ChIP, Lot D2816, RRID:AB_737197

Mouse monoclonal anti-V5, Cat#R960-25, Invitrogen (ThermoFisher), co-IP/WB, Lot 1869182, RRID:AB_2556564

Mouse monoclonal anti-V5-HRP, Cat#R961-25, Invitrogen (ThermoFisher), WB, Lot 2084452, RRID:AB_2556565

Mouse monoclonal anti-ERG, Cat#sc-376293, Santa Cruz Biotechnology ,WB , Lot C0218, RRID:AB_10989086

Mouse monoclonal anti- HNF-1B, Cat#sc-130407, Santa Cruz Biotechnology, WB, Lot E2119, RRID:AB_2248215

Rabbit monoclonal IgG, Cat#ab172730, Abcam, ChIP/co-IP, Lot GR3235749-21, RRID:AB_2687931

Rabbit monoclonal anti-ERG, Cat#ab92513, Abcam, ChIP/co-IP/WB, Lot GR219881-32, RRID:AB_2630401

Mouse monoclonal anti-FLAG, Cat#F1804, Sigma-aldrich, co-IP/WB, Lot SLBK1346V, RRID:Addgene_86282

Goat anti-mouse IgG (H+L) secondary antibody HRP, Cat#32430, Thermo Fisher, WB, Lot LK152904, RRID:AB_1185566

Goat anti-rabbit IgG (H+L) secondary antibody HRP, Cat#32460, Thermo Fisher, WB , Lot UH287786, RRID:AB_1185567

Anti-rabbit Androgen Receptor, Cat#ab108341, Abcam, ChIP, Lot GR3233428-4, RRID:AB_10865716

H3K4me1, Cat#ab8895, Abcam, ChIP,Lot GR3293005-1, RRID:AB_306847

H3K4me2, Cat#39141 , ACTIVE MOTIF, ChIP,Lot 1008001, RRID:AB_2614985

H3K4me3, Cat#ab12209, Abcam, ChIP, Lot GR3253794-3, RRID:AB_442957

H3K27ac, Cat#ab4729 , Abcam, ChIP, Lot GR3357415-1, RRID:AB_2118291

Mouse polyclonal IgG Cat#sc-2025, Santa Cruz Biotechnology,ChIP, Lot J1518, RRID:AB_737182

Diluted and used methods:
For western blot assay:
Rabbit polyclonal anti-HNF1B, 1:1000 (Ab µl: blocking buffer µl)
Mouse monoclonal anti-HNF-1B, 1:1000 (Ab µl: blocking buffer µl)
Mouse monoclonal anti-FLAG, 1:1000 (Ab µl: blocking buffer µl)
Mouse monoclonal anti-V5, 1:5000 (Ab µl: blocking buffer µl)
Mouse monoclonal anti-V5-HRP, 1:5000 (Ab µl: blocking buffer µl)
Mouse monoclonal anti-ERG, 1:5000 (Ab µl: blocking buffer µl)
Rabbit monoclonal anti-ERG, 1:5000 (Ab µl: blocking buffer µl)
Goat anti-rabbit IgG secondary antibody, 1:5000 (Ab µl: blocking buffer µl)
Goat anti-mouse IgG secondary antibody, 1:5000 (Ab µl: blocking buffer µl)

For Co-Immunoprecipitation assay:
mouse monoclonal anti-V5, 5µg
rabbit monoclonal IgG, 5µg
rabbit monoclonal anti-ERG, 5µg
mouse monoclonal anti-FLAG, 5µg

For ChIP assay:
Rabbit polyclonal anti- HNF1B, 8µg
Rabbit polyclonal IgG, 8µg
Rabbit monoclonal IgG, 8µg
Mouse polyclonal IgG, 8µg
Rabbit monoclonal anti-ERG, 8µg
Anti-rabbit Androgen Receptor, 8µg
H3K4me1, 8µg
H3K4me2, 8µg
H3K4me3, 8µg
H3K27ac, 8µg

Validation

Rabbit polyclonal anti- HNF1B Cat#sc-22840X
https://www.scbt.com/p/hnf-1beta-antibody-h-85
Citation: HNF-1β Regulates Transcription of the PKD Modifier Gene Kif12
Yimei Gong, Zhendong Ma, Vishal Patel, Evelyne Fischer, Thomas Hiesberger, Marco Pontoglio, Peter Igarashi
JASN Jan 2009, 20 (1) 41-47; DOI: 10.1681/ASN.2008020238
Tchorz JS, Kinter J, Müller M, Tornillo L, Heim MH, Bettler B. Notch2 signaling promotes biliary epithelial cell fate specification and tubulogenesis during bile duct development in mice. Hepatology. 2009 Sep;50(3):871-9. doi: 10.1002/hep.23048. PMID: 19551907.

Rabbit polyclonal IgG Cat#sc-2027X
https://datasheets.scbt.com/sc-2027.pdf
Citation: Mal A, Sturniolo M, Schiltz RL, Ghosh MK, Harter ML. A role for histone deacetylase HDAC1 in modulating the transcriptional activity of MyoD: inhibition of the myogenic program. EMBO J. 2001 Apr 2;20(7):1739-53. doi: 10.1093/emboj/20.7.1739. PMID: 11285237; PMCID: PMC145490.

Mouse monoclonal anti-V5 Cat#R960-25
https://www.thermofisher.com/antibody/product/V5-Tag-Antibody-Monoclonal/R960-25
Validation statement of manufacturer: ''This antibody is functionally tested against 20 ng of an E. coli expressed fusion protein containing a V5 epitope using a chemiluminescent substrate at a 1 minute exposure. This antibody has also been tested in Western blot against 25 ng of recombinant Positope™ protein.''
Citation:Hansen AS, Hsieh TS, Cattoglio C, Pustova I, Saldaña-Meyer R, Reinberg D, Darzacq X, Tjian R. Distinct Classes of Chromatin Loops Revealed by Deletion of an RNA-Binding Region in CTCF. Mol Cell. 2019 Nov 7;76(3):395-411.e13. doi: 10.1016/j.molcel.2019.07.039. Epub 2019 Sep 12. PMID: 31522987; PMCID: PMC7251926.
Chai N, Haney MS, Couthouis J, Morgens DW, Benjamin A, Wu K, Ousey J, Fang S, Finer S, Bassik MC, Gitler AD. Genome-wide synthetic lethal CRISPR screen identifies FIS1 as a genetic interactor of ALS-linked C9ORF72. Brain Res. 2020 Feb 1;1728:146601. doi: 10.1016/j.brainres.2019.146601. Epub 2019 Dec 13. PMID: 31843624; PMCID: PMC7539795.

Mouse monoclonal anti-V5-HRP Cat#R961-25
https://www.thermofisher.com/antibody/product/V5-Tag-Antibody-Monoclonal/R961-25
Validation statement of manufacturer: ''V5-His-LacZ was detected at ~117 kDa using V5-HRP Mouse Monoclonal Antibody (Product # R961-25) at 1:3000 dilution in 2.5% skim milk at 4°C overnight on a rocking platform. Chemiluminescent detection was performed using Pierce™ ECL Western Blotting Substrate (Product # 32106).''
Citation:Zhang C, Chen Y, Sun S, Zhang Y, Wang L, Luo Z, Liu M, Dong L, Dong N, Wu Q. A conserved LDL-receptor motif regulates corin and CD320 membrane targeting in polarized renal epithelial cells. Elife. 2020 Nov 2;9:e56059. doi: 10.7554/eLife.56059. PMID: 33136001; PMCID: PMC7605860.

Mouse monoclonal anti-ERG Cat#sc-376293
https://www.scbt.com/p/erg-1-2-3-antibody-c-1
Validation statement of manufacturer: Showing figure, western blot results. ''Western blot analysis of Erg-1/2/3 expression in CCRF-CEM whole cell lysate.''
Citation: Alholle, A., Karanian, M., Brini, A.T., Morris, M.R., Kannappan, V., Niada, S., Niblett, A., Ranchère-Vince, D., Pissaloux, D., Delfour, C., Maran-Gonzalez, A., Antonescu, C.R., Sumathi, V., Tirode, F. and Latif, F. (2018), Genetic analyses of undifferentiated small round cell sarcoma identifies a novel sarcoma subtype with a recurrent CRTC1-SS18 gene fusion. J. Pathol, 245: 186-196. https://doi.org/10.1002/path.5071

Mouse monoclonal anti- HNF-1B Cat#sc-130407
https://www.scbt.com/p/hnf-1beta-antibody-94-8
Validation statement of manufacturer: Showing figure, western blot results. ''Western blot analysis of HNF-1β expression in human skin tissue extract.''
Citation: Sun Y, Wang X, Bu X. LINC02381 contributes to cell proliferation and hinders cell apoptosis in glioma by transcriptionally enhancing CBX5. Brain Res Bull. 2021 Nov;176:121-129. doi: 10.1016/j.brainresbull.2021.07.009. Epub 2021 Jul 15. PMID: 34274429.

Rabbit monoclonal IgG Cat#ab172730
https://www.abcam.com/rabbit-igg-monoclonal-epr25a-isotype-control-ab172730.html
Validation statement of manufacturer: Showing figure, western blot results. ''Rabbit monoclonal IgG (ab172730) instead of ab124962 in NIH/3T3 whole cell lysate.''
Citation: Zhou, J, Zhou, H, Liu, C, Huang, L, Lu, D, Gao, C. HDAC1-mediated deacetylation of LSD1 regulates vascular calcification by promoting autophagy in chronic renal failure. J Cell Mol Med. 2020; 24: 8636– 8649. https://doi.org/10.1111/jcmm.15494.

Rabbit monoclonal anti-ERG Cat#ab92513

https://www.abcam.com/erg-antibody-epr3864-ab92513.html
Validation statement of manufacturer: Showing figure, western blot results. ''Anti-ERG antibody [EPR3864] (ab92513) at 1/1000 dilution (unpurified) + Jurkat (human T cell leukemia cell line from peripheral blood) cell lysate at 10 µg.''
Citation: Andrade J, Shi C, Costa ASH, Choi J, Kim J, Doddaballapur A, Sugino T, Ong YT, Castro M, Zimmermann B, Kaulich M, Guenther S, Wilhelm K, Kubota Y, Braun T, Koh GY, Grosso AR, Frezza C, Potente M. Control of endothelial quiescence by FOXO-regulated metabolites. Nat Cell Biol. 2021 Apr;23(4):413-423. doi: 10.1038/s41556-021-00637-6. Epub 2021 Apr 1. PMID: 33795871; PMCID: PMC8032556.

Mouse monoclonal anti-FLAG Cat#F1804
https://www.sigmaaldrich.com/FI/en/product/sigma/f1804
Citation: Qiu Y, Liu P, Ma X, Ma X, Zhu L, Lin Y, You Y, Yu W, Ma D, Sun C, Qin Z, Zhao Y, Shi J, Han L. TRIM50 acts as a novel Src suppressor and inhibits ovarian cancer progression. Biochim Biophys Acta Mol Cell Res. 2019 Sep;1866(9):1412-1420. doi: 10.1016/j.bbamcr.2019.06.002. Epub 2019 Jun 6. PMID: 31176697.

https://www.encodeproject.org/antibodies/ENCAB697XQW/

H3K4me1 Cat#ab8895
https://www.abcam.com/histone-h3-mono-methyl-k4-antibody-chip-grade-ab8895.html
Validation statement of manufacturer: Showing figure''Chromatin was prepared from U-2 OS cells according to the Abcam X-ChIP protocol. Cells were fixed with formaldehyde for 10min. The ChIP was performed with 25µg of chromatin, 2µg of ab8895 (blue), and 20µl of Protein A/G sepharose beads.''
Citation:Parsa S, Ortega-Molina A, Ying HY, Jiang M, Teater M, Wang J, Zhao C, Reznik E, Pasion JP, Kuo D, Mohan P, Wang S, Camarillo JM, Thomas PM, Jain N, Garcia-Bermudez J, Cho BK, Tam W, Kelleher NL, Socci N, Dogan A, De Stanchina E, Ciriello G, Green MR, Li S, Birsoy K, Melnick AM, Wendel HG. The serine hydroxymethyltransferase-2 (SHMT2) initiates lymphoma development through epigenetic tumor suppressor silencing. Nat Cancer. 2020;1:653-664. doi: 10.1038/s43018-020-0080-0. Epub 2020 Jun 22. PMID: 33569544; PMCID: PMC7872152.

https://www.encodeproject.org/antibodies/ENCAB249ROX/

H3K4me2 Cat#39141
https://www.activemotif.com/catalog/details/39141/histone-h3-dimethyl-lys4-antibody-pab#image_1
Validation statement of manufacturer: ''This antibody has been validated for use in ChIP and/or ChIP-Seq, and can be used with Active Motif's ChIP-IT® High Sensitivity Kit or our magnetic bead-based ChIP-IT® Express Kits.''
Citation:Serebryannyy, L.A., et. al. 2019. "HiPLA: High-throughput imaging proximity ligation assay." Methods. Mar 15;157:80-87. (Proximity Ligation Assay)

https://www.encodeproject.org/antibodies/ENCAB346FTT/

H3K4me3 Cat#ab12209
https://www.abcam.com/histone-h3-tri-methyl-k4-antibody-mabcam12209-chip-grade-ab12209.html
Validation statement of manufacturer: ''Chromatin was prepared from U2OS cells according to the Abcam X-ChIP protocol. Cells were fixed with formaldehyde for 10min. The ChIP was performed with 25 µg of chromatin, 2 µg of ab12209 (blue), and 20 µl of Protein A/G sepharose beads.''
Citation:Arumugam, T.; Ghazi, T.; Chuturgoon, A. Fumonisin B1 Epigenetically Regulates PTEN Expression and Modulates DNA Damage Checkpoint Regulation in HepG2 Liver Cells. Toxins 2020, 12, 625. https://doi.org/10.3390/toxins12100625.

https://www.encodeproject.org/antibodies/ENCAB000BKY/

H3K27ac Cat#ab4729
https://www.abcam.com/histone-h3-acetyl-k27-antibody-chip-grade-ab4729.html
Validation statement of manufacturer: ''Chromatin was prepared from HeLa (Human epithelial cell line from cervix adenocarcinoma) cells according to the Abcam X-ChIP protocol. Cells were fixed with formaldehyde for 10 minutes. The ChIP was performed with 25 µg of chromatin, 2 µg of ab4729 (blue), and 20 µl of Protein A/G sepharose beads.''
Citation: Lu B, He Y, He J, Wang L, Liu Z, Yang J, Gao Z, Lu G, Zou C, Zhao W. Epigenetic Profiling Identifies LIF as a Super-enhancer-Controlled Regulator of Stem Cell-like Properties in Osteosarcoma. Mol Cancer Res. 2020 Jan;18(1):57-67. doi: 10.1158/1541-7786.MCR-19-0470. Epub 2019 Oct 15. PMID: 31615908.

https://www.encodeproject.org/antibodies/ENCAB000APD/

Mouse polyclonal IgG Cat#sc-2025
https://www.scbt.com/p/normal-mouse-igg
Citation:Trivedi A, Mehrotra A, Baum CE, Lewis B, Basuroy T, Blomquist T, Trumbly R, Filipp FV, Setaluri V, de la Serna IL. Bromodomain and extra-terminal domain (BET) proteins regulate melanocyte differentiation. Epigenetics Chromatin. 2020 Mar 10;13(1):14. doi: 10.1186/s13072-020-00333-z. PMID: 32151278; PMCID: PMC7063807.

https://www.encodeproject.org/antibodies/ENCAB000AOG/

Anti-rabbit Androgen Receptor Cat#ab108341
https://www.abcam.com/androgen-receptor-antibody-er1792-chip-grade-ab108341.html
Validation statement of manufacturer: ''Chromatin was prepared from LNCaP cells according to the Abcam Dual-X-ChIP protocol*. Cells were fixed with 1.5 mM EGS for 30mins and then formaldehyde for 10min.
The ChIP was performed with 25 µg of chromatin, 5 µg of ab108341 (red), or 5 µg of rabbit normal IgG ab172730 (gray) and 20 µl of Protein A/G sepharose beads.''
Citation:Zhao J, Zhang Y, Liu XS, Zhu FM, Xie F, Jiang CY, Zhang ZY, Gao YL, Wang YC, Li B, Xia SJ, Han BM. RNA-binding protein

Musashi2 stabilizing androgen receptor drives prostate cancer progression. Cancer Sci. 2020 Feb;111(2):369-382. doi: 10.1111/cas.14280. Epub 2020 Jan 4. PMID: 31833612; PMCID: PMC7004550.

Eukaryotic cell lines

Policy information about cell lines

Cell line source(s)

22Rv1	ATCC	Cat#CRL-2505 https://www.atcc.org/products/crl-2505
LNCaP	ATCC	Cat#CRL-1740 https://www.atcc.org/products/crl-1740
RWPE1	ATCC	Cat#CRL-11609 https://www.atcc.org/products/crl-11609
VCaP	ATCC	Cat#CRL-2876 https://www.atcc.org/products/crl-2876
293T	ATCC	Cat#CRL-11268 https://www.atcc.org/products/crl-11268
A549	ATCC	Cat#CCL-185 https://www.atcc.org/products/ccl-185
DU145	ATCC	Cat#HTB-81 https://www.atcc.org/products/htb-81
PC3	ATCC	Cat#CRL-1435 https://www.atcc.org/products/crl-1435
V16A	Hua et al. Cell 2018	Citation: Hua JT, Ahmed M, Guo H, Zhang Y, Chen S, Soares F, Lu J, Zhou S, Wang M, Li H, Larson NB, McDonnell SK, Patel PS, Liang Y, Yao CQ, van der Kwast T, Lupien M, Feng FY, Zoubeidi A, Tsao MS, Thibodeau SN, Boutros PC, He HH. Risk SNP-Mediated Promoter-Enhancer Switching Drives Prostate Cancer through lncRNA PCAT19. Cell. 2018 Jul 26;174(3):564-575.e18. doi: 10.1016/j.cell.2018.06.014. Epub 2018 Jul 19. PMID: 30033362.

Authentication

The cell lines have not been authenticated. Most of cells lines were purchased by ATCC. Cell morphology and growth rate of the cell lines used in this study were similar to previous reports. These cell lines have been used in previous publications.

Mycoplasma contamination

Cells were negative after tested for mycoplasma contamination.

Commonly misidentified lines
(See ICLAC register)

No commonly misidentified cell lines were used.

Palaeontology and Archaeology

Specimen provenance

Provide provenance information for specimens and describe permits that were obtained for the work (including the name of the issuing authority, the date of issue, and any identifying information). Permits should encompass collection and, where applicable, export.

Specimen deposition

Indicate where the specimens have been deposited to permit free access by other researchers.

Dating methods

If new dates are provided, describe how they were obtained (e.g. collection, storage, sample pretreatment and measurement), where they were obtained (i.e. lab name), the calibration program and the protocol for quality assurance OR state that no new dates are provided.

☐ Tick this box to confirm that the raw and calibrated dates are available in the paper or in Supplementary Information.

Ethics oversight

Identify the organization(s) that approved or provided guidance on the study protocol, OR state that no ethical approval or guidance was required and explain why not.

Note that full information on the approval of the study protocol must also be provided in the manuscript.

Animals and other organisms

Policy information about studies involving animals; ARRIVE guidelines recommended for reporting animal research

Laboratory animals

For laboratory animals, report species, strain, sex and age OR state that the study did not involve laboratory animals.

Wild animals

Provide details on animals observed in or captured in the field; report species, sex and age where possible. Describe how animals were caught and transported and what happened to captive animals after the study (if killed, explain why and describe method; if released, say where and when) OR state that the study did not involve wild animals.

Field-collected samples

For laboratory work with field-collected samples, describe all relevant parameters such as housing, maintenance, temperature, photoperiod and end-of-experiment protocol OR state that the study did not involve samples collected from the field.

Ethics oversight

Identify the organization(s) that approved or provided guidance on the study protocol, OR state that no ethical approval or guidance was required and explain why not.

Note that full information on the approval of the study protocol must also be provided in the manuscript.

Human research participants

Policy information about studies involving human research participants

Population characteristics

Describe the covariate-relevant population characteristics of the human research participants (e.g. age, gender, genotypic information, past and current diagnosis and treatment categories). If you filled out the behavioural & social sciences study design questions and have nothing to add here, write "See above."

Recruitment	*Describe how participants were recruited. Outline any potential self-selection bias or other biases that may be present and how these are likely to impact results.*
Ethics oversight	*Identify the organization(s) that approved the study protocol.*

Note that full information on the approval of the study protocol must also be provided in the manuscript.

Clinical data

Policy information about clinical studies

All manuscripts should comply with the ICMJE guidelines for publication of clinical research and a completed CONSORT checklist must be included with all submissions.

Clinical trial registration	*Provide the trial registration number from ClinicalTrials.gov or an equivalent agency.*
Study protocol	*Note where the full trial protocol can be accessed OR if not available, explain why.*
Data collection	*Describe the settings and locales of data collection, noting the time periods of recruitment and data collection.*
Outcomes	*Describe how you pre-defined primary and secondary outcome measures and how you assessed these measures.*

Dual use research of concern

Policy information about dual use research of concern

Hazards

Could the accidental, deliberate or reckless misuse of agents or technologies generated in the work, or the application of information presented in the manuscript, pose a threat to:

No	Yes	
☒	☐	Public health
☒	☐	National security
☒	☐	Crops and/or livestock
☒	☐	Ecosystems
☒	☐	Any other significant area

Experiments of concern

Does the work involve any of these experiments of concern:

No	Yes	
☒	☐	Demonstrate how to render a vaccine ineffective
☒	☐	Confer resistance to therapeutically useful antibiotics or antiviral agents
☒	☐	Enhance the virulence of a pathogen or render a nonpathogen virulent
☒	☐	Increase transmissibility of a pathogen
☒	☐	Alter the host range of a pathogen
☒	☐	Enable evasion of diagnostic/detection modalities
☒	☐	Enable the weaponization of a biological agent or toxin
☒	☐	Any other potentially harmful combination of experiments and agents

ChIP-seq

Data deposition

☒ Confirm that both raw and final processed data have been deposited in a public database such as GEO.

☒ Confirm that you have deposited or provided access to graph files (e.g. BED files) for the called peaks.

Data access links<br>*May remain private before publication.*	The HNF1B ChIP-seq data have been deposited in the European Nucleotide Archive (ENA) under accessions PRJEB46088. The ChIPseq data for HNF1B and ERG replicates are available at ENA under accession PRJEB49662. BED files are provided in the Source Data.
Files in database submission	PRJEB46088:<br>ERR6183026 HNF1B DHT VCaP raw<br>ERR6183027 Input DHT VCaP raw<br>ERR6183028 HNF1B DHT VCaP processed

ERR6183029	Input DHT VCaP processed
PRJEB49662:	
ERR7815565	ERG DHT VCaP raw
ERR7815567	INPUT DHT VCaP raw
ERR7815568	ERG ETH VCaP raw
ERR7815569	HNF1B ETH VCaP raw
ERR7815570	INPUT ETH VCaP raw
ERR7815571	ERG DHT VCaP processed
ERR7815573	INPUT DHT VCaP processed
ERR7815574	ERG ETH VCaP processed
ERR7815575	HNF1B ETH VCaP processed
ERR7815576	INPUT ETH VCaP processed

Genome browser session (e.g. UCSC)

no longer applicable

Methodology

Replicates

Two biological replicate per sample

Sequencing depth

Each sample in the first study sequenced 13-19M single-end reads. Each sample in the replicate study produced 86-107M single-end reads.

Antibodies

Rabbit polyclonal anti- HNF1B, Cat#sc-22840X , ChIP-seq, Santa Cruz Biotechnology, F3016
Rabbit monoclonal anti-ERG, Cat#ab92513,ChIP-seq, Abcam, GR219881-32
Rabbit polyclonal IgG, Cat#sc-2027X, Santa Cruz Biotechnology, ChIP-seq, D2816

Peak calling parameters

The ChIP-seq peaks were called using MACS2 v2.2.7.1 with default settings.

Data quality

Data quality was measured by FastQC and further controlled by Trimmomatic with parameters: TruSeq2-SE.fa:2:30:10 SLIDINGWINDOW:5:20

Software

Software listed above, described in the methods section of the manuscript.

Flow Cytometry

Plots

Confirm that:

☐ The axis labels state the marker and fluorochrome used (e.g. CD4-FITC).

☐ The axis scales are clearly visible. Include numbers along axes only for bottom left plot of group (a 'group' is an analysis of identical markers).

☐ All plots are contour plots with outliers or pseudocolor plots.

☐ A numerical value for number of cells or percentage (with statistics) is provided.

Methodology

Sample preparation

Describe the sample preparation, detailing the biological source of the cells and any tissue processing steps used.

Instrument

Identify the instrument used for data collection, specifying make and model number.

Software

Describe the software used to collect and analyze the flow cytometry data. For custom code that has been deposited into a community repository, provide accession details.

Cell population abundance

Describe the abundance of the relevant cell populations within post-sort fractions, providing details on the purity of the samples and how it was determined.

Gating strategy

Describe the gating strategy used for all relevant experiments, specifying the preliminary FSC/SSC gates of the starting cell population, indicating where boundaries between "positive" and "negative" staining cell populations are defined.

☐ Tick this box to confirm that a figure exemplifying the gating strategy is provided in the Supplementary Information.

Magnetic resonance imaging

Experimental design

Design type

Indicate task or resting state; event-related or block design.

Design specifications

Specify the number of blocks, trials or experimental units per session and/or subject, and specify the length of each trial or block (if trials are blocked) and interval between trials.

Behavioral performance measures

State number and/or type of variables recorded (e.g. correct button press, response time) and what statistics were used to establish that the subjects were performing the task as expected (e.g. mean, range, and/or standard deviation across subjects).

Acquisition

Imaging type(s)

Specify: functional, structural, diffusion, perfusion.

Field strength

Specify in Tesla

Sequence & imaging parameters

Specify the pulse sequence type (gradient echo, spin echo, etc.), imaging type (EPI, spiral, etc.), field of view, matrix size, slice thickness, orientation and TE/TR/flip angle.

Area of acquisition

State whether a whole brain scan was used OR define the area of acquisition, describing how the region was determined.

Diffusion MRI ☐ Used ☐ Not used

Preprocessing

Preprocessing software

Provide detail on software version and revision number and on specific parameters (model/functions, brain extraction, segmentation, smoothing kernel size, etc.).

Normalization

If data were normalized/standardized, describe the approach(es): specify linear or non-linear and define image types used for transformation OR indicate that data were not normalized and explain rationale for lack of normalization.

Normalization template

Describe the template used for normalization/transformation, specifying subject space or group standardized space (e.g. original Talairach, MNI305, ICBM152) OR indicate that the data were not normalized.

Noise and artifact removal

Describe your procedure(s) for artifact and structured noise removal, specifying motion parameters, tissue signals and physiological signals (heart rate, respiration).

Volume censoring

Define your software and/or method and criteria for volume censoring, and state the extent of such censoring.

Statistical modeling & inference

Model type and settings

Specify type (mass univariate, multivariate, RSA, predictive, etc.) and describe essential details of the model at the first and second levels (e.g. fixed, random or mixed effects; drift or auto-correlation).

Effect(s) tested

Define precise effect in terms of the task or stimulus conditions instead of psychological concepts and indicate whether ANOVA or factorial designs were used.

Specify type of analysis: ☐ Whole brain ☐ ROI-based ☐ Both

Statistic type for inference
(See Eklund et al. 2016)

Specify voxel-wise or cluster-wise and report all relevant parameters for cluster-wise methods.

Correction

Describe the type of correction and how it is obtained for multiple comparisons (e.g. FWE, FDR, permutation or Monte Carlo).

Models & analysis

n/a	Involved in the study
☐	☐ Functional and/or effective connectivity
☐	☐ Graph analysis
☐	☐ Multivariate modeling or predictive analysis

Functional and/or effective connectivity

Report the measures of dependence used and the model details (e.g. Pearson correlation, partial correlation, mutual information).

Graph analysis

Report the dependent variable and connectivity measure, specifying weighted graph or binarized graph, subject- or group-level, and the global and/or node summaries used (e.g. clustering coefficient, efficiency, etc.).

Multivariate modeling and predictive analysis

Specify independent variables, features extraction and dimension reduction, model, training and evaluation metrics.
